# Deciphering Water Oxidation Catalysts: The Dominant Role of Surface Chemistry over Reconstruction Degree in Activity Promotion

**DOI:** 10.1007/s40820-024-01562-7

**Published:** 2024-11-26

**Authors:** Li An, Jianyi Li, Yuanmiao Sun, Jiamin Zhu, Justin Zhu Yeow Seow, Hong Zhang, Nan Zhang, Pinxian Xi, Zhichuan J. Xu, Chun-Hua Yan

**Affiliations:** 1 State Key Laboratory of Applied Organic Chemistry, Key Laboratory of Nonferrous Metal Chemistry and Resources Utilization of Gansu Province, Frontiers Science Center for Rare Isotopes, College of Chemistry and Chemical Engineering, Lanzhou University, Lanzhou, 730000 People’s Republic of China; 2 School of Materials Science and Engineering, Nanyang Technological University, Singapore, 639798 Singapore; 3 Faculty of Materials Science and Energy Engineering, Institute of Technology for Carbon Neutrality, Shenzhen Institute of Advanced Technology, Chinese Academy of Sciences, Shenzhen, 518055 People’s Republic of China; 4 Key Laboratory of Electromagnetic Materials and Devices, National Center for International Research On Photoelectric and Energy Materials, School of Materials and Energy, Electron Microscopy Center, Yunnan University, Kunming, 650091 People’s Republic of China; 5 State Key Laboratory of Baiyunobo Rare Earth Resource Researches and Comprehensive Utilization, Baotou Research Institute of Rare Earths, Baotou, 014030 People’s Republic of China; 6 Beijing National Laboratory for Molecular Sciences, State Key Laboratory of Rare Earth Materials Chemistry and Applications, PKU-HKU Joint Laboratory in Rare Earth Materials and Bioinorganic Chemistry, Peking University, Beijing, 100871 People’s Republic of China

**Keywords:** Oxygen evolution reaction, Perovskite oxides, Doping, Activation and reconstruction

## Abstract

Water splitting hinges crucially on the availability of electrocatalysts for the oxygen evolution reaction. The surface reconstruction has been widely observed in perovskite catalysts, and the reconstruction degree has been often correlated with the activity enhancement. Here, a systematic study on the roles of Fe substitution in activation of perovskite LaNiO_3_ is reported. The substituting Fe content influences both current change tendency and surface reconstruction degree. LaNi_0.9_Fe_0.1_O_3_ is found exhibiting a volcano-peak intrinsic activity in both pristine and reconstructed among all substituted perovskites in the LaNi_1-x_Fe_x_O_3_ (*x* = 0.00, 0.10, 0.25, 0.50, 0.75, 1.00) series. The reconstructed LaNi_0.9_Fe_0.1_O_3_ shows a higher intrinsic activity than most reported NiFe-based catalysts. Besides, density functional theory calculations reveal that Fe substitution can lower the O 2*p* level, which thus stabilize lattice oxygen in LaNi_0.9_Fe_0.1_O_3_ and ensure its long-term stability. Furthermore, it is vital interesting that activity of the reconstructed catalysts relied more on the surface chemistry rather than the reconstruction degree. The effect of Fe on the degree of surface reconstruction of the perovskite is decoupled from that on its activity enhancement after surface reconstruction. This finding showcases the importance to customize the surface chemistry of reconstructed catalysts for water oxidation.

**Supplementary Information:**

The online version contains supplementary material available at 10.1007/s40820-024-01562-7.

## Highlights

- Demonstrate that the key factor to determine the activity of reconstructed surfaces is the surface chemistry, instead of reconstruction degree using the popular perovskite LaNi_1-x_Fe_x_O_3_ oxides as model materials.
- Fe content can influence both the surface reconstruction degree, the activation degree, and the activity of reconstructed surfaces.
- The oxygen evolution reaction activity of reconstructed catalysts is primarily governed by the chemistry of the reconstructed surface species.

**Supplementary Information:**

The online version contains supplementary material available at 10.1007/s40820-024-01562-7.

## Graphical abstract

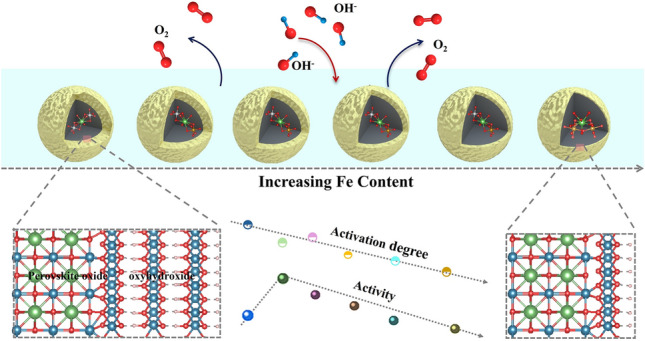

## Introduction

The increasing demands of global energy consumption call for a sustainable and environmentally friendly energy supply [1]. Hydrogen offers one of the most promising solutions because of its zero-carbon attribute [2]. Electrochemical water splitting is a clean and direct approach to producing green hydrogen, provided that the electrolyzer can be powered by electricity obtained from renewable sources [3, 4]. However, the efficiency of water splitting is technically hindered by the sluggish oxygen evolution reaction (OER) at the anode, which necessitates the design of efficient OER electrocatalysts [5, 6].

Transition metal perovskite oxides have been demonstrated recently as a class of materials capable to efficiently catalyze OER in alkaline condition [7]. The ABO_3_ crystal frame of perovskites enables the A and B sites to be resided by alkali/alkaline-earth/rare earth metals and first-row transition metals, respectively. Within the structure, the A- and/or B-site cations can be substituted by a foreign cation having different radius or oxidation state [8]. Therefore, the composition and cation oxidation state of perovskites are highly manipulable, which offers a feasible and convenient way to correlate the physicochemical properties with the catalytic performance of perovskite-type oxides, LaNiO_3_ is a well-known conducting material (ρ = 9 × 10^–3^ Ω cm) at room temperature because of the stable low-spin (LS) t_2g_^6^e_g_^1^ configuration of Ni^3+^(d^7^) [9–11]. It has been found that OER catalyzed by LaNiO_3_, especially by the compressively strained LaNiO_3_, prefers to proceed via lattice oxygen mechanism (LOM) rather than adsorbate evolution mechanism (AEM) [12, 13]. The activation of lattice oxygen in LOM implies that the structure of perovskite LaNiO_3_ may easily undergo dynamic in-situ structural reconstruction under OER conditions. More recently, it has been reported that the structure of nickel-based transition metal oxides (TMOs) can easily transform into oxyhydroxides after long-term electrochemical cycling, enabling the in-situ generated NiOOH to function as the real active species for OER [14].

However, the OER activity of pure NiOOH is found to be relatively poor. It has been intensively observed that the presence of Fe in nickel-based oxyhydroxides, either directly incorporated in the pre-catalyst or accidentally deposited from electrolyte solution, can effectively improve the catalytic activity of the electrode [15–19]. Although it remains debatable whether nickel or iron cations serve as the active centers, the optimal metallic fraction of iron in nickel–iron oxyhydroxide is found to be less than 30% (nickel higher than 70%) [20]. Meanwhile, the degree to which the original perovskite phase can reconstruct during OER also contribute to the measured activity by influencing the number of available active sites. The reconstruction behavior is highly sensitive to the bulk chemistry of perovskite. It is critical to identify the optimal nickel–iron ratio in perovskite pre-catalyst to optimize its degree of reconstruction and subsequent oxyhydroxide OER activity [21].

In this work, using perovskite LaNi_1-x_Fe_x_O_3_ (*x* = 0.00, 0.10, 0.25, 0.50, 0.75, and 1.00, which was simplified as LaNi_1-x_Fe_x_O_3_ below) as model catalysts, we investigate the role of iron cations during surface reconstruction and reveal the optimal nickel–iron ratio in the perovskite pre-catalyst. Our results show that the activity of the reconstructed LaNi_0.9_Fe_0.1_O_3_ outperforms other perovskites, and its intrinsic activity is even higher than some benchmark NiFe-based catalysts. The low level of Fe substitution in LaNiO_3_ obviously accelerates the reconstruction rate and enables enhanced activity in both its pristine and reconstructed state. However, since reconstructed LaNiO_3_ with the maximum reconstruction degree exhibits lower OER activity than reconstructed LaNi_0.9_Fe_0.1_O_3_, the effect of Fe doping on the degree of surface reconstruction of the perovskite is effectively decoupled from that on its OER activity enhancement after electrochemical treatment. Unveiled by density functional theory (DFT) calculations, LaNi_0.9_Fe_0.1_O_3_/Ni_0.9_Fe_0.1_OOH with balanced reconstruction degree and reconstruction rate was demonstrated as the most stable interface structure. Besides, Fe substitution lowers the O 2*p* level and thus stabilizes the lattice oxygen in LaNi_0.9_Fe_0.1_O_3_. As a result, Fe incorporation creates more stable surface chemistry to provide enhanced structural stability compared with pure LaNiO_3._ The surface amorphous layer composed of Ni_0.9_Fe_0.1_OOH with atomic Fe incorporated Ni sites as active sites showing obvious electronic activity can further ensure enhanced activity and long-term stability under OER conditions.

## Experimental Section

### Materials

Lanthanum (III) nitrate hexahydrate (La(NO_3_)_3_·6H_2_O, 99.9%), nickel (II) acetate hexahydrate (Ni(OAc)_2_·6H_2_O, 99.0%), iron (III) nitrate nonahydrate (Fe(NO_3_)_3_·9H_2_O, 98.5%), urea (CO(NH_2_)_2_, 99.0%), citric acid monohydrates (C_6_H_8_O_7_·H_2_O, 99.5%), potassium hydroxide (KOH, 99.9%), and Nafion® (5 wt%) were purchased from Aladdin. The deionized (DI) water was obtained from a Millipore Autopure system (18.2 MΩ, Millipore Ltd., USA). Oxygen gas was of 5 N quality (99.999%, Airgas). All the other materials for electrochemical measurements were of analytical grade and were used without further purification.

### Preparation of LaNi_1-x_Fe_x_O_3_

LaNi_1-x_Fe_x_O_3_ powders were synthesized by a sol–gel method. The LaNiO_3_ precursors were produced by dissolving 3.75 mmol La(NO_3_)_3_·6H_2_O, 3.75 mmol Ni(OAc)_2_·6H_2_O, 15 mmol urea, 15 mmol citric acids in 30 mL of deionized (DI) water and 3 mL of nitric acid. As for LaNi_1-x_Fe_x_O_3_, Fe(NO_3_)_3_·9H_2_O and Ni(OAc)_2_·6H_2_O were added stoichiometrically for Fe to replace Ni. LaFeO_3_ precursor was prepared by using 3.75 mmol Fe(NO_3_)_3_·9H_2_O instead of Ni(OAc)_2_·6H_2_O. Increasing the heating temperature to 150 °C with a stable magnetic stirring until the above precursor solutions become a gel state. Then, the gel was dried at 170 °C for 12 h to remove the remaining water and calcined at 600 °C in the O_2_ atmosphere for 6 h.

### Electrochemical Measurements

The as-synthesized LaNi_1-x_Fe_x_O_3_ perovskite oxide (16 mg) was mixed with acetylene black (6.4 mg). The mixture was then dispersed in a mixed solution of water (6.4 mL), isopropanol (1.6 mL), and Nafion solution (100 μL) to prepare the homogeneous ink of the catalyst. The obtained catalyst ink was drop-casted on glassy carbon electrode (GC, with diameter of 5 mm) with a geometric area of 0.196 cm^2^. The final mass loading of catalyst is 255 μg_ox_ cm^−2^_disk_.

All electrochemical measurements were performed at room temperature on a Biologic SP-150 workstation (Bio-Logic Science Instruments) using a conventional three-electrode setup. For OER in media (1.0 M KOH), a Pt plate and mercury-mercury oxide electrode (Hg/HgO) electrode (filled with 1.0 M KOH solution) were used as counter and reference electrodes, respectively. The reference electrode was calibrated against reversible hydrogen electrode (RHE) in H_2_-saturated electrolyte with Pt plates as both the working and counter electrodes, which should be consistent with the Eq. (1):

1 $${\text{E}}\left( {{\text{vs}}.{\text{ RHE}}} \right) = {\text{E}}\left( {{\text{vs}}.{\text{ Hg}}/{\text{HgO}}} \right) + 0.0{98} + 0.0{59} \times {\text{pH}}$$

Before the electrochemical measurement, the KOH electrolyte was degassed by bubbling oxygen for at least 30 min to obtain the oxygen gas saturated condition. All electrochemical experiments were conducted at 20 ± 0.2 °C. Cyclic voltammetry (CV) was set at the alternating scan rates of 10 mV s^−1^ and 100 mV s^−1^. Here, we used the data of the 2nd and 5000th cycles (without further obvious current increase in the subsequent cycles) to represent pre-catalysts and reconstructed catalysts respectively for characterization and analysis. These cycles were collected at a scan rate of 10 mV s^−1^. For the intermediate cycles, a scan rate of 100 mV s^−1^ was applied. Tafel slopes were obtained by averaging the positive-going and negative-going scans of CV curves obtained with the scan rate of 10 mV s^−1^. These data were collected and corrected for the uncompensated (iR) contribution within the cell through Eq. (2):

2 $${\text{Ereal}} = {\text{E}}\left( {{\text{vs}}.{\text{ RHE}}} \right){-}{\text{iR}}$$

For the analysis of the correlations between reconstruction degree, activation degree and OER activity, reconstruction degree and activation degree have been defined as follow Eq. (3):

3 $${\text{Reconstruction degree }} = {\text{ t/1 nm}}$$

where *t* represents the thickness of the newly formed reconstructed surface layer.

Activation degree = Δ*j*/*j*_as-synthesized_, where Δ*j* represents the change in current density after activation relative to current density before activation (*j*_reconstructed_−*j*_as-synthesized_).

### Computational Details

The Vienna Ab-initio Simulation Package (VASP) was carried out for the LaNi_1-x_Fe_x_O_3_ system calculations [55]. The Perdew–Burke–Ernzerhof (PBE) functional, within the generalized gradient approximation (GGA) approach, was used to describe the exchange and correlation effects. For all the geometry optimizations, the cutoff energy was set to 450 eV. The force and energy convergence tolerance were set to be 0.05 eV Å^−1^ and 10^–5^ eV, respectively. The models of LaNi_1-x_Fe_x_O_3_ (*x* = 0.00, 0.10, 0.25, 0.50, 0.75, and 1.00) supercells were constructed by replacing Fe atoms with Ni atoms in a LaFeO_3_ structure. The Monkhorst–Pack k-point mesh was set to be 6 × 6 × 2 and 6 × 6 × 4 for performing calculations on LaNi_1-x_Fe_x_O_3_ (*x* = 0.00, 0.10, 0.25, 0.50, 0.75, 1.00). The Hubbard U corrections were adopted using the model proposed by Dudarev and colleagues. The Uef (Uef = Coulomb (U)–exchange (J)) values of Ni and Fe were set to be 6.4 and 4 eV, respectively. The pure heterostructure is composed of three amorphous NiOOH layers and two crystalline LaNiO_3_ layers. The Fe doped only has two NiFeOOH layers. The molecular dynamics simulation was adopted to calculate the amorphous structure for 1.5 ps with a time step of 1 fs. The GGA-PBE functional is selected for the exchange and correlation potential [56]. Weak van der Waals interaction is considered by the DFT-D_3_ functional [57]. The cut off energy for the plane-wave is 400 eV. The Gamma point in the Brillouin-zone is chosen for integration. Total energies of the systems converge to 10^–5^ eV in the iteration solution of Kohn–Sham equation. The force on each atom reduces to 0.05 eV Å^−1^ after geometry optimization. The amorphous structure is built by ab-initio molecular dynamics simulation using Nosé-Hoover thermostat for 1.7 ps with a time step of 1 fs [58]. The crystalline structure is melted at 3500 K for 0.6 ps, which was then quenched to the room temperature (300 K) for 1.1 ps.

## Results and Discussion

### Crystal and Electronic Structure Analysis

**Fig. 1:**
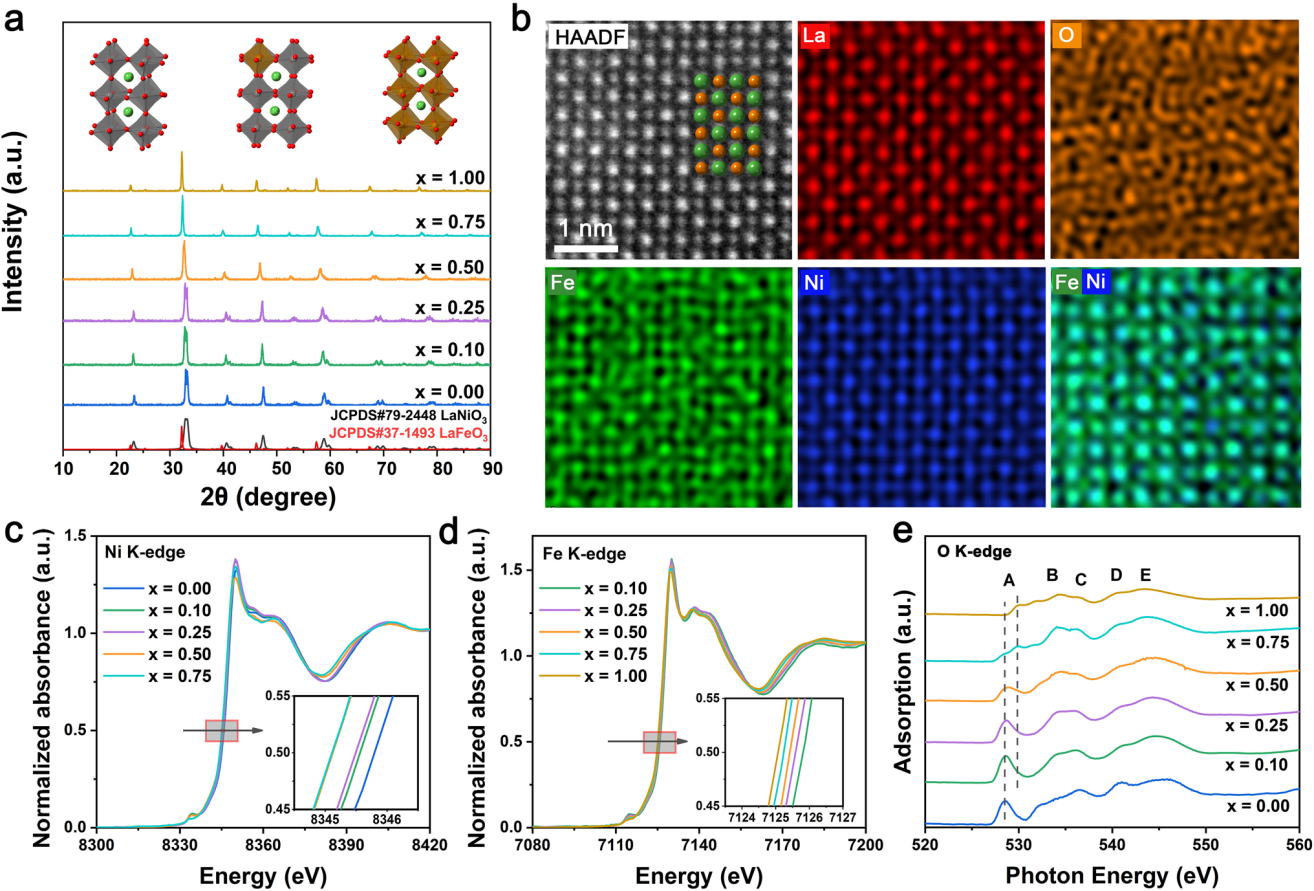
**a** XRD patterns of LaNi_1-x_Fe_x_O_3_ in comparison with the standard XRD patterns of LaNiO_3_ (JCPDS 79-2448) and LaFeO_3_ (JCPDS 37-1493). **b** Atomic-resolution HAADF-STEM image and corresponding EDS atomic elemental mapping images of LaNi_0.9_Fe_0.1_O_3_. Inset shows the crystal structure scheme of LaNi_0.9_Fe_0.1_O_3_ proposed based on the HAADF-STEM image, where yellow spheres represent La cations and red spheres represent Ni/Fe cations. **c** Normalized Ni K-edge XANES spectra for LaNi_1-x_Fe_x_O_3_ (*x* = 0.00, 0.10, 0.25, 0.50, 0.75). **d** Normalized Fe K-edge XANES spectra for LaNi_1-x_Fe_x_O_3_ (*x* = 0.10, 0.25, 0.50, 0.75, 1.00). Insets provide the magnified images of Ni K-edge XANES spectra and Fe K-edge XANES spectra. **e** O K-edge XAS spectra of LaNi_1-x_Fe_x_O_3_

Perovskite LaNi_1-x_Fe_x_O_3_ with different Fe contents was prepared using a sol–gel method [22]. Demonstrated by the inductively coupled plasma optical emission spectroscopy (ICP-OES), the actual amounts of Fe in the prepared system are quite close to that in the nominal stoichiometric composition (Table S1). The crystal structures of the LaNi_1-x_Fe_x_O_3_ oxides were characterized by powder X-ray diffraction (XRD). As shown in Figs.1a and S1, no diffraction peaks of La_2_O_3_ and NiO can be found, demonstrating the formation of pure LaNi_1-x_Fe_x_O_3_ oxide series without impurity. LaNi_0.5_Fe_0.5_O_3_ exhibits a mixture of both the rhombohedral and orthorhombic structures. Furthermore, with more Ni being replaced by Fe (0.10 < *x* < 1.00), an obvious diffraction peak shift toward a smaller angle is observed (Fig.S1). Such peak shift is ascribed to lattice expansion induced by the difference in ionic radii between Ni and Fe cations, which indicates the successful substitution of Ni by Fe [23, 24]. Field-emission scanning electron microscope (FE-SEM) images (Fig. S2) illustrate that all the as-synthesized LaNi_1-x_Fe_x_O_3_ adopt similar morphology of irregular shapes. The transmission electron microscopy (TEM) image (Fig. S3) and the corresponding energy-dispersive spectrum (EDS) elemental mappings confirm the successful synthesis of LaNi_0.9_Fe_0.1_O_3_ and La, Ni, Fe, and O are uniformly distributed in the entire architecture (Fig. S4). The atomic-resolution high-angle annular dark-field aberration-corrected scanning transmission electron microscopy (HAADF-STEM) image and corresponding EDS atomic elemental mappings were then carried out to disclose the position of the doped Fe in the LaNiO_3_ host. As shown in Figs. 1b and S5, all the atoms are uniformly arranged and the doped Fe atoms display the same arrangement as Ni atoms, revealing the similar site geometrical occupation of Fe and Ni. Collectively, we can conclude that Fe doping into pure LaNiO_3_ perovskite has resulted in only substitution of Ni ions [25]. This phenomenon can be rationalized by the electronic interaction between the host Ni and the dopant Fe, which thus modulates the local atomic coordination environment of NiO_6_ motifs at B sites in perovskite structures [26].

It is observed that the majority valence states of nickel and iron cations are + 3 in pure LaNiO_3_ and LaFeO_3_ conducted by X-ray photoelectron spectroscopy (XPS). However, small amounts of Ni^2+^ and Fe^2+^ are detected (Fig. S6), indicating the existence of oxygen vacancies on the surface [23, 27, 28]. With increasing Fe content in LaNi_1-x_Fe_x_O_3_ (*x* = 0.00, 0.10, 0.25, 0.50, 0.75), Ni^2+^/Ni^3+^ ratio increases (Fig. S7), demonstrating that introducing Fe into the LaNi_1-x_Fe_x_O_3_ system reduces the oxidation state of Ni [29, 30]. The subtle changes of local bonding environments and coordination structures were investigated by X-ray absorption spectroscopy (XAS) [31]. The absorption edge energies were defined by the half-height approach [32, 33]. From Fig. 1c and d, absorption edge energies of both Ni and Fe decrease with Fe doping, further demonstrating the reduced oxidation states of metals and the interaction between host Ni and dopant Fe. The coordination environment and symmetry of metal centers can also be analyzed from the pre-edge feature of the metal K-edge peak (Fig. S8, Note S1). Compared with pure LaFeO_3_, the more pronounced pre-edge peak (1*s* → 3*d* state transition) of LaNi_1-x_Fe_x_O_3_ (*x* = 0.10, 0.25, 0.50, 0.75) suggests more distorted lattice arrangement [26, 34]. The lattice distortion may act as the structural origin for the dynamic structural evolution of LaNi_1-x_Fe_x_O_3_ (*x* = 0.10, 0.25, 0.50, 0.75) during electrochemical process. The Fourier transform (FT) of k_3_-weighted extended X-ray absorption fine structure (EXAFS) characterization (Fig. S9) and the corresponding wavelet transformation (WT) analysis (Fig. S10) were further used to demonstrate the strong interaction between metals Ni and Fe. Besides, O K-edge XAS was also utilized to study the electronic structure of LaNi_1-x_Fe_x_O_3_. The special peak A in the spectra can be attributed to the band hybridization between O 2*p* and M (Ni/Fe) 3*d*. The obvious positive shift of peak A with increasing Fe doping in LaNiO_3_ further supports the inference of decreased metals valence after introducing Fe (Figs. 1e and S11) [27, 33, 35–37]. Electron energy loss spectroscopy (EELS) was performed on selected samples to further understand their electronic structures. For Ni L-edge and Fe L-edge, the negative shift of Ni L_3_ from *x* = 0 to *x* = 0.10 and the reduced Fe L_3_/L_2_ ratio from *x* = 1.00 to *x* = 0.10 confirm the interaction between Ni and Fe (Fig. S12a and b). As observed by O K-edge of EELS, the disappearance of pre-peak located at around 531.5 eV indicates the decreased hybridization between O 2*p* and M 3*d* or an increase in oxygen vacancies (Fig. S12c). Owing to the changed oxidation states of Ni and Fe, the O 2*p*-M 3*d* hybridization can be excluded, therefore the pre-peak disappearance in O K-edge EELS spectra is attributed to the formation of oxygen vacancies [35–38]. Besides, EELS spectra of Ni L-edge and Fe L-edge were obtained from scans on LaNi_0.9_Fe_0.1_O_3_ along the direction shown in Fig. S13. Compared with the stable peaks of Fe–L_3_, the obvious positive shift of La-M_4_/Ni-L_3_ convoluted peak from Point 1 to Point 7 demonstrates that the oxidation state of outer Ni is much lower than that of inner Ni. This observation implies that the introduction of Fe causes the formation of oxygen vacancies in LaNi_0.9_Fe_0.1_O_3_, which mainly exist at the surface of perovskite oxides (Fig. S14) [27]. Collectively, we can reasonably conclude that without damaging the main crystal structure in the bulk phase, surface oxygen vacancies and lattice distortions created by appropriate Fe substitution increase the flexibility of the perovskite structure, which can facilitate structural evolution under electrochemical treatment.

### Electrochemical Characterizations and Activation Degree Analysis

**Fig. 2:**
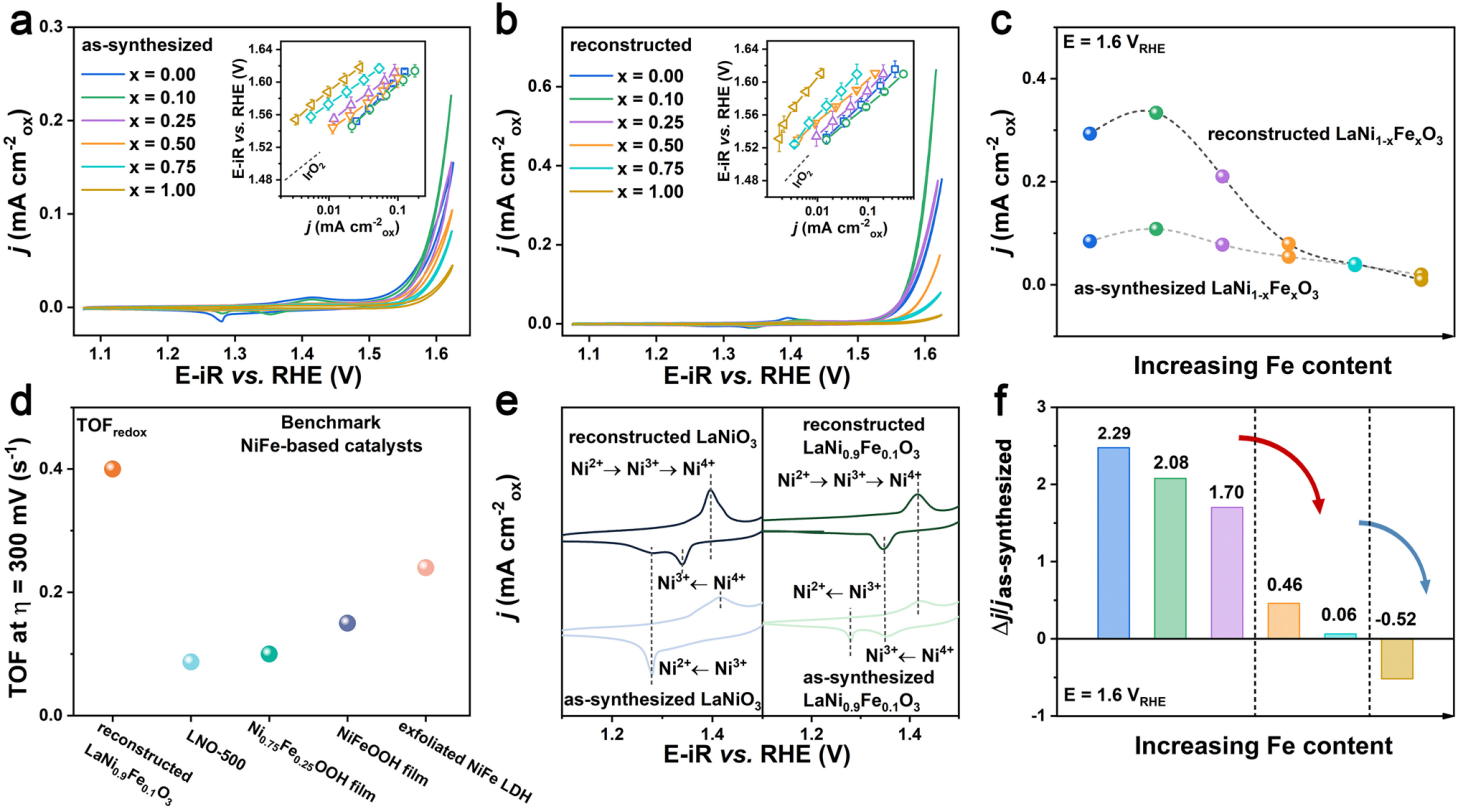
**a** iR-corrected cyclic voltammograms (CVs) of as-synthesized LaNi_1-x_Fe_x_O_3_. **b** iR-corrected CVs of reconstructed LaNi_1-x_Fe_x_O_3_. Both sets of CVs were obtained during measurement in O_2_-saturated 1.0 M KOH (99.999%) at a scan rate of 10 mV s^−1^. Insets present the corresponding Tafel plots normalized by BET surface areas of (*A*) as-synthesized and (*B*) reconstructed LaNi_1-x_Fe_x_O_3_ compared with IrO_2_ [41]. Each error bar represents standard deviation of three independent measurements. **c** Surface area-normalized current densities (*j*) of the as-synthesized and reconstructed LaNi_1-x_Fe_x_O_3_ at 1.6 V. **d** Turnover frequency (TOF) of the reconstructed LaNi_0.9_Fe_0.1_O_3_ at an overpotential (*η*) of 300 mV. The number of active sites for TOF calculation is estimated by the coulombic charge under the M redox peak (TOF_redox_) (see Note S3). **e** Evolution of redox behavior of representative LaNiO_3_ (left) and LaNi_0.9_Fe_0.1_O_3_ (right) perovskite oxides in 1.0 M KOH at 10 mV s^−1^ between 1.1 and 1.5 V. **f** Relative current change of LaNi_1-x_Fe_x_O_3_, where the current densities were obtained at 1.6 V. All potential data are iR-corrected and Δ*j* represents the change in current

The effect of Fe doping in perovskite oxides was further evaluated by their electrocatalytic performance. All potential data are converted to the reversible hydrogen electrode (RHE) scale for direct comparison. To avoid the possibility of Fe-impurity-induced activity enhancement, Fe-free electrolyte prepared from electronic grade KOH (99.999% purity) was used for the OER characterizations, detailed procedures of which are provided in Note S2. Figure 2a and b shows the cyclic voltammetry (CV) curves (normalized to the Brunauer–Emmett–Teller (BET) surface area, as shown in Table S2) of the as-synthesized and surface-reconstructed LaNi_1-x_Fe_x_O_3_, respectively. We can see similar activity trends with LaNi_0.9_Fe_0.1_O_3_ showing the best intrinsic activity. These trends are attributed to the introduction of Fe, which is consistent with recent reports that excess iron content would lead to Fe phase segregation and subsequent activity degradation [39, 40]. After surface reconstruction, the current density at 1.6 V of LaNi_0.9_Fe_0.1_O_3_ increases and becomes comparable to that of the benchmark IrO_2_ [41] in the OER regime (Fig. 2c). This indicates a surface reconstruction of the crystalline LaNi_0.9_Fe_0.1_O_3_ that improved OER activity [42]. Moreover, by normalizing the activity with the number of redox-active sites, the intrinsic activity of reconstructed LaNi_0.9_Fe_0.1_O_3_, expressed as turnover frequency (TOF, see Note S3) at an OER overpotential (η) of 300 mV, was compared with those of the state-of-the-art NiFe-based electrocatalysts in Fig. 2d. It is observed that after surface activation, the intrinsic TOF of reconstructed LaNi_0.9_Fe_0.1_O_3_ becomes superior to that of LaNiO_3_-500 perovskite oxide [38], Ni_0.75_Fe_0.25_OOH film [16], NiFeOOH film [43], and exfoliated NiFe layered double hydroxides (LDH) [44], manifesting the excellent OER activity of the reconstructed LaNi_0.9_Fe_0.1_O_3_. This activation process can also be observed in the chronopotentiometry (CP) of pristine LaNi_0.9_Fe_0.1_O_3_ at a current density of 10 mA cm^−2^ for about 20 h, which also shows excellent stability with a negligible potential increase over the next 80 h of the durability test (Fig. S15). This 100 h CP test also demonstrates the stability performance of LaNi_0.9_Fe_0.1_O_3_. Furthermore, the almost unchanged morphology and structure observed from the XRD pattern and TEM images after long-term reaction suggest the robust structure stability of LaNi_0.9_Fe_0.1_O_3_ (Fig. S16). Thus, LaNi_0.9_Fe_0.1_O_3_ after activation has demonstrated as an ideal OER catalyst in terms of both activity and stability.

The reduction–oxidation behavior of LaNi_1-x_Fe_x_O_3_ perovskite oxides can be studied through cyclic voltammograms of the pristine oxides and surface-reconstructed catalytic surfaces displayed in Figs. S17 and S18. Generally, for *x* ≤ 0.50, the redox peak areas and OER activities increase after the OER activation process, demonstrating the gradual activation of metal active sites underneath the pristine oxide surface [17, 45]. The subsequent surface reconstruction correlates with oxidation processes in the pre-OER regime [8, 46], which is sensitive to the Ni/Fe ratio of LaNi_1-x_Fe_x_O_3_ oxides. The subsequent surface reconstruction correlates with oxidation processes in the pre-OER regime [8, 46], which is sensitive to the Ni/Fe ratio of LaNi_1-x_Fe_x_O_3_ oxides. For detailed analysis, we take LaNiO_3_ and LaNi_0.9_Fe_0.1_O_3_ as our models for comparison. Compared with the as-synthesized LaNiO_3_ that has only one clear pair of redox peaks, two reduction peaks and one oxidation peak can be observed for reconstructed LaNiO_3_, in which the reduction peaks at ~ 1.34 and ~ 1.28 V are assigned to Ni(IV)/Ni(III) and Ni(III)/Ni(II), respectively (Fig. 2e). This suggests the presence of two reversible redox reactions; thus we believe that the anodic peak at ~ 1.39 V should be the result of the convolution of oxidation peaks attributed to Ni(II)/Ni(III) and Ni(III)/Ni(IV) transitions. For the as-synthesized LaNi_0.9_Fe_0.1_O_3_, it possesses redox features similar to reconstructed LaNiO_3_ (i.e., two resolved reduction peaks and one convoluted oxidation peak). However, the reduction peak of Ni(III)/Ni(II) disappears for reconstructed LaNi_0.9_Fe_0.1_O_3_, demonstrating a much more stable Ni^3+^ state in LaNi_0.9_Fe_0.1_O_3_ than in LaNiO_3_. Besides, the Ni oxidation peaks shift toward higher potentials upon Fe incorporation (Fig. S19), which can be used as an indicator of the presence of Fe in the main structure as demonstrated by many reports [25, 26]. This suggests a strong synergistic electronic interaction between Ni and Fe [47]. Meanwhile, the reduction peaks of Ni(IV)/Ni(III) also show a slight anodic shift upon the addition of Fe. These results imply that while it is more difficult to oxidize Ni^3+^ to Ni^4+^ in the presence of Fe, Ni^4+^ is more easily to be reduced to Ni^3+^ in reconstructed LaNi_0.9_Fe_0.1_O_3_. Then, combined with the disappearance of Ni^2+^ after the activation process, we can conclude that incorporating Fe can facilitate Ni^3+^ to reach dynamic equilibrium for constructing a stable active layer in the presence of Ni^3+^ species. When looking into pure LaFeO_3_, we notice that the areas for both the oxidation and reduction peaks (at ~ 1.38 and ~ 1.26 V, respectively) are much smaller than that of LaNiO_3_ (Fig. S20a and b), which can be overlooked in comparison with the CV curves of LaNi_0.9_Fe_0.1_O_3_ [48]. Besides, the redox peaks of LaFeO_3_ remain almost stable before and after reconstruction (Fig. S20c). Thus, we believe the reconstruction behavior is mainly contributed by Ni. Furthermore, we also estimated the charge involved in both the oxidation and reduction peaks of the LaNi_0.9_Fe_0.1_O_3_ (Fig. S21; see Note S4) to study the redox of surface-active sites and their OER activity [18, 49]. The increased redox charge after activation (Table S3) suggests the enhanced oxidability/reducibility and intrinsic activity for reconstructed LaNi_0.9_Fe_0.1_O_3_ oxide. We then attribute the related OER activity enhancement after surface reconstruction to the increment in the number of active sites [1], owing to the newly reconstructed surface.

By comparing average current densities (averaging the positive-going and negative-going scans),  LaNi_0.5_Fe_0.5_O_3_ and LaNi_0.25_Fe_0.75_O_3_ show only slight positive current change. For pure LaFeO_3_, it yields directly decreased OER current during the activation process (Fig. S22). It is found that LaNiO_3_ possesses the highest activation degree, and the substitution of Fe can inhibit this process with LaFeO_3_ even showing a negative value (Fig. 2f). However, LaNiO_3_ shows lower OER activity than LaNi_0.9_Fe_0.1_O_3_. These results confirm the role of Fe substitution in controlling the activity and activation degree of LaNiO_3_.

### Surface Reconstruction Degree and Surface Reconstruction Rate Analysis

**Fig. 3:**
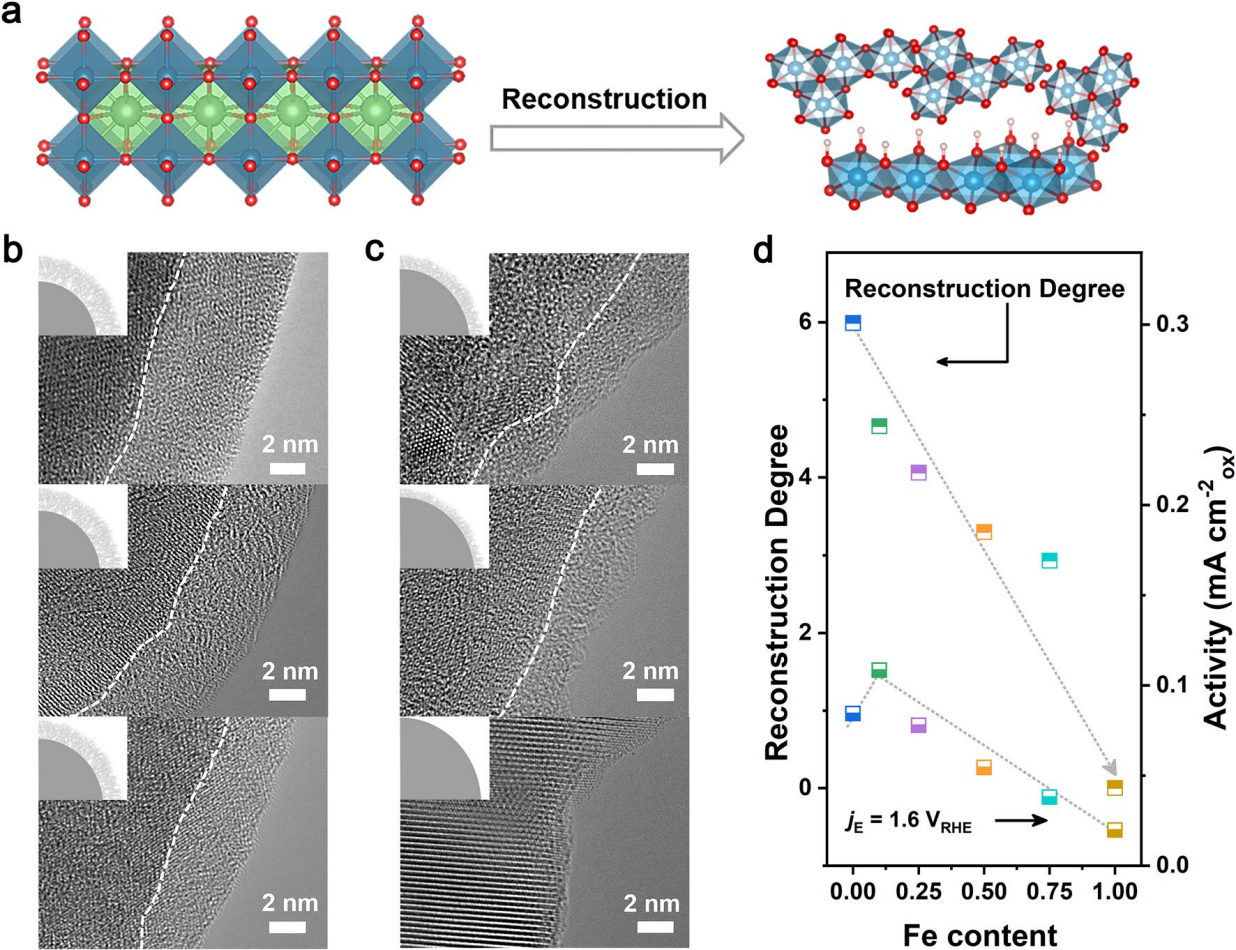
**a** Schematic diagrams of the crystal structures before and after reconstruction. **b** The HRTEM images of the reconstructed LaNiO_3_, LaNi_0.9_Fe_0.1_O_3_, and LaNi_0.75_Fe_0.25_O_3_ (from up to down). **c** HRTEM images of the reconstructed LaNi_0.5_Fe_0.5_O_3_, LaNi_0.25_Fe_0.75_O_3_, and LaFeO_3_ (from up to down). **d** Current densities at 1.6 V and the thickness of the newly formed reconstructed surface layer for LaNi_1-x_Fe_x_O_3_

Compared with the crystalline as-obtained LaNi_0.9_Fe_0.1_O_3_ (Fig. S23), a newly formed amorphous layer without long-range order is observed by high-resolution transmission electron microscopy (HRTEM) on the surface of reconstructed LaNi_0.9_Fe_0.1_O_3_ (Fig. S24). This reconstruction process is shown in Fig. 3a. Without changing the crystal structure of the bulk phase as evidenced by XRD (Fig. S16), the depth for the outermost amorphous surface stays at 4.5 nm (Fig. S25). This thickness is demonstrated as optimal active surface depth for in-situ formed oxyhydroxides active species, which has been widely reported and accepted [50]. Meanwhile, we used EELS to investigate the reconstructed surface layer by obtaining an energy loss profile across the surface-bulk interface from Point 1 to Point 7 (Fig. S26a). Interestingly, compared with the profile obtained for the as-synthesized LaNi_0.9_Fe_0.1_O_3_, the pre-edge feature at around 532 eV of the reconstructed LaNi_0.9_Fe_0.1_O_3_ disappears at the surface region (Fig. S26b and c), suggesting an increase in oxygen vacancies on the surface. This evidence can further support the formation of amorphous surface layers after surface reconstruction. Fourier transform infrared (FTIR) spectra in Fig. S27 confirm the presence of MOOH structure by exhibiting a peak at ~ 1230 cm^−1^ after CV activation [51]. Thus, the element composition of this active surface layer is deduced as amorphous NiFe (oxy)hydroxide with abundant oxygen deficiencies. As shown in the XPS results, surface Ni and Fe cations exhibit higher oxidation states after activation (Fig. S28). The ICP-OES data further demonstrated that the overall Ni:Fe ratio shows no obvious change before (8:95:1.00) and after reconstruction (8:75:1.00). The kinetically stabilized Ni^3+^ due to Fe doping would induce a strong surface hydroxylation of the reconstructed LaNi_0.9_Fe_0.1_O_3_ oxide and thus lead to the formation of highly active Ni_0.9_Fe_0.1_OOH to serve as the true reaction site for OER. For reconstructed LaNi_0.9_Fe_0.1_O_3_ with active Ni_0.9_Fe_0.1_OOH surface after 100 h CP measurement, the similar amorphous layer thickness of about 4.5 nm further indicates the stable structure of the reconstructed layer (Figs. S29 and S30). The short-range amorphization on the surface ensures the long-time stability of reconstructed LaNi_0.9_Fe_0.1_O_3_ oxide because this amorphous layer might impede the proton transport into the bulk perovskite structure. In addition, DFT calculations demonstrate that the interface structure of LaNi_0.9_Fe_0.1_O_3_/Ni_0.9_Fe_0.1_OOH with lower binding energy (−80.32 eV) is much more stable than that of the LaNiO_3_/NiOOH (−79.59 eV), further keeping the reconstructed LaNi_0.9_Fe_0.1_O_3_ an ideal durability (Fig. S31). Besides, it is worth noting that the thickness of newly formed reconstructed surface layers gradually decreases along with the increment of Fe content in the LaNi_1-x_Fe_x_O_3_ series from 5.99 to 2.93 nm (*x* = 0.00, 0.10, 0.25, 0.50, 0.75) and even without obvious reconstruction layer for pure LaFeO_3_ (Fig. 3b and c). Such a negative correlation demonstrates that Fe incorporation can suppress the surface reconstruction of LaNi_1-x_Fe_x_O_3_ perovskite oxide. Moreover, the thickness (t) of the reconstructed surface layer or the reconstruction degree (t /1 nm) shows a similar trend consistent with the activation degree (Fig. 2f) of LaNi_1-x_Fe_x_O_3_, while not consistent with their OER activity (Fig. 3d).

**Fig. 4:**
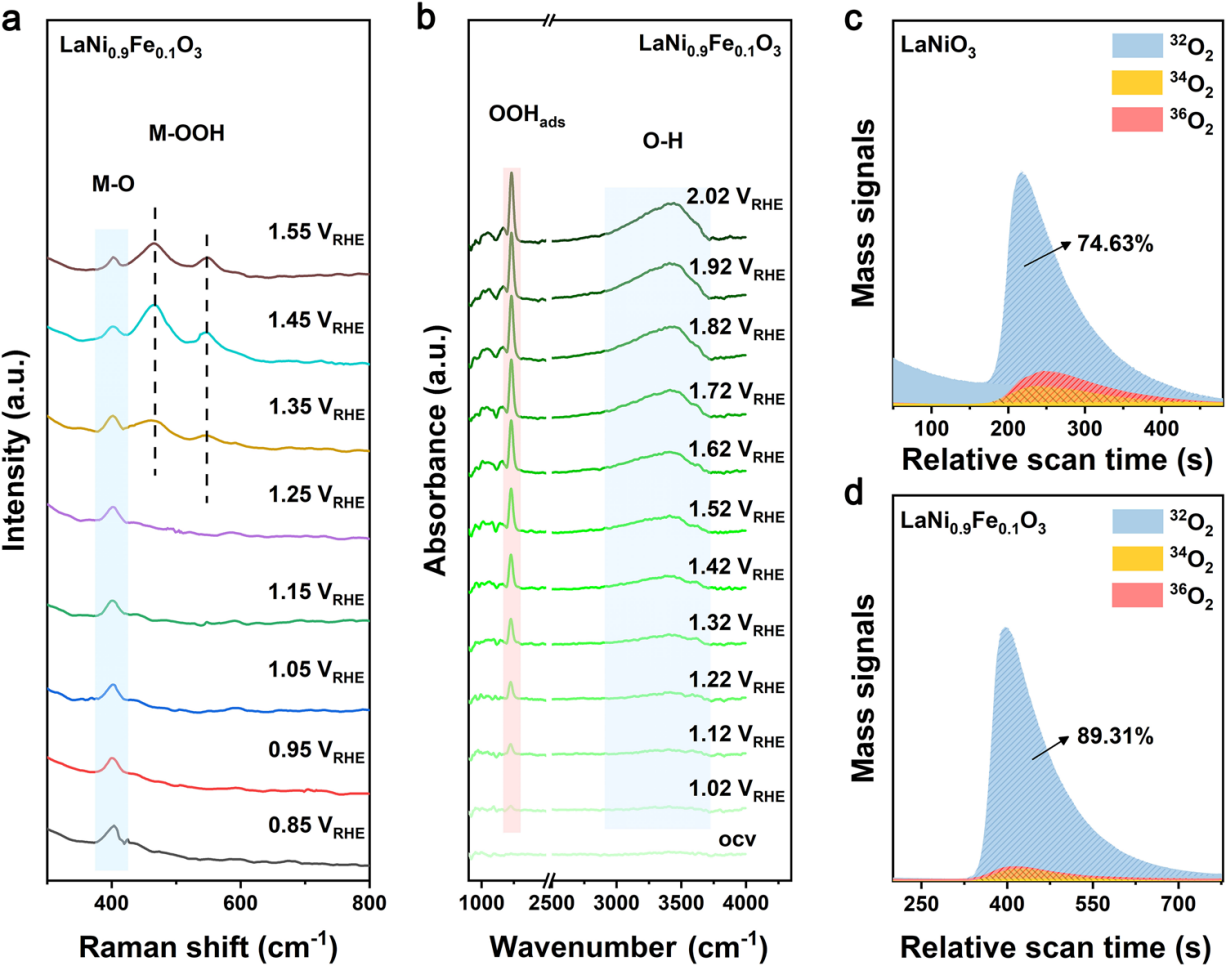
**a** Potential-dependent in-situ Raman spectra of LaNi_0.9_Fe_0.1_O_3_. **b** In-situ ATR-IR spectra recorded during the multi-potential steps for LaNi_0.9_Fe_0.1_O_3_. **c** DEMS signals of ^32^O_2_ (^16^O^16^O), and ^34^O_2_ (^16^O^18^O) & ^36^O_2_ (^18^O^18^O) from the reaction products for ^18^O-labeled LaNiO_3_. **d** DEMS signals of ^32^O_2_ (^16^O^16^O), and ^34^O_2_ (^16^O^18^O) & ^36^O_2_ (^18^O^18^O) from the reaction products for.^18^O-labeled LaNi_0.9_Fe_0.1_O_3_

Potential-dependent in-situ Raman spectroscopy was further performed to monitor the dynamic surface reconstruction. In the obtained Raman spectra (Figs. 4a and S32a), the peaks at ~ 400 cm^−1^ are attributed to M–O bonds [52]. At this Raman shift, LaNiO_3_ exhibits two peaks, while LaNi_0.9_Fe_0.1_O_3_ shows only one. This is owing to their different inner structure induced by the incorporation of Fe, which results in different rates and degrees of surface reconstruction. The gradual emerging peaks at 460 and 550 cm^−1^ indicate the surface change from perovskite oxide to oxyhydroxide (MOOH) [52]. It should be noted that the peak of MOOH appears at 1.55 V for LaNiO_3_ while such appearance occurred at 1.35 V for LaNi_0.9_Fe_0.1_O_3_, indicating that Fe doping can accelerate the formation of oxyhydroxide layers. This is further illustrated by in-situ attenuated total reflection infrared (ATR-IR) spectra. As displayed in Figs. 4b and S32b, both the ATR-IR spectra of LaNiO_3_ and LaNi_0.9_Fe_0.1_O_3_ show two peaks from 900 to 4350 cm^−1^. The peak at ~ 1200 cm^−1^ is associated with the O–O stretching mode of surface-adsorbed superoxide (OOH_ads_), which is a typical reaction intermediate of AEM; while the adsorption band located at around 3400 cm^−1^ is attributed to surface-adsorbed OH species [53]. It is found that OH species appeared at a lower potential of 1.02 V for LaNi_0.9_Fe_0.1_O_3_ compared with pure LaNiO_3_ (1.22 V). This is consistent with the result from in-situ Raman spectra; that is, the incorporation of Fe can increase the reconstruction rate during the OER process. Besides, the emerged OOH_ads_ for LaNi_0.9_Fe_0.1_O_3_ demonstrates that the lattice oxygen oxidation process (which corresponds to lattice oxygen mechanism, LOM) is suppressed after incorporating Fe compared to LaNiO_3_. ^18^O-labeled LaNiO_3_ and LaNi_0.9_Fe_0.1_O_3_ were analyzed by in-situ differential electrochemical mass spectroscopy (DEMS) to track the OER process in ^16^O KOH electrolyte. It is noteworthy that compared with the (^36^O_2_ + ^34^O_2_)/^32^O_2_ ratio of LaNiO_3_ (34%), the ratio for LaNi_0.9_Fe_0.1_O_3_ is greatly decreased to 12% (Figs. 4c and d and S33). This obvious reduction indicates that the LOM process is greatly suppressed by Fe substitution, thus leading to a limited surface reassembly degree and enhanced structural stability. These results indicate that Fe (*x* = 0.1) doping accelerates the rate of surface reconstruction and promotes the formation of a stable surface structure prohibiting continuous reconstruction, thus leading to a reduced degree of reconstruction and enhanced structural stability.

### Activation Degree, Reconstruction Degree, and Intrinsic Activity Analysis

**Fig. 5:**
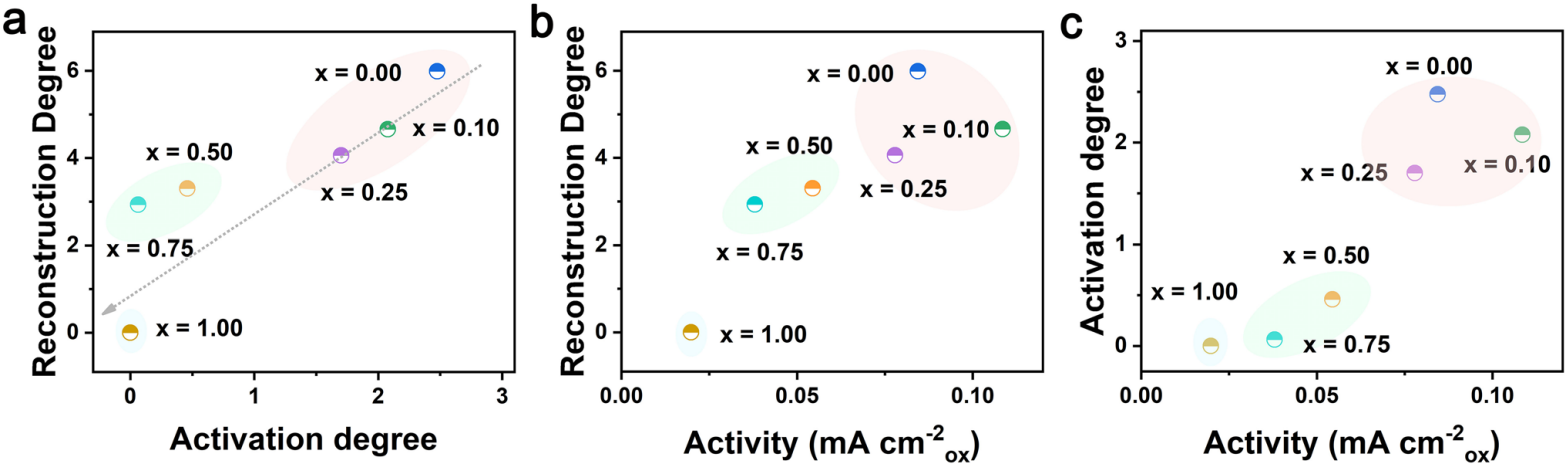
**a** Relationship between activation degree and reconstruction degree. **b** Relationship between activity and reconstruction degree. **c** Relationship between activity and activation degree. The activation degree of LaFeO_3_ is deemed as 0, because of its decreased OER current after surface reconstruction

In this study, we use the relative current change after surface reconstruction and the thickness of amorphous surface layers as measurable proxies for activation degree and reconstruction degree, respectively. To investigate the effect of electrochemical active surface formation, the relationship between activation degree, reconstruction degree (as defined in Methods), and OER activity of LaNi_1-x_Fe_x_O_3_ are presented in Fig. 5. It is found that reconstruction degree is proportional to activation degree as both the reconstruction degree and activation degree gradually decrease along with the increment of Fe content in the LaNi_1-x_Fe_x_O_3_ (Fig. 5a). This result demonstrates that the Fe doping can suppress the surface reconstruction and inhibit the activation degree of LaNi_1-x_Fe_x_O_3_. However, as displayed in Fig. 5b and c, no meaningful correlation can be derived between OER activity and either the reconstruction degree or activation degree. Reconstructed LaNiO_3_ with the maximum reconstruction degree and largest activation degree shows lower OER activity than reconstructed LaNi_0.9_Fe_0.1_O_3_. We thus believe that Fe doping can affect the OER activity of LaNi_1-x_Fe_x_O_3_. Besides, different Ni/Fe ratio can also affect reconstruction degree and activation degree for LaNi_1-x_Fe_x_O_3_. It has been widely accepted that the surface reconstruction of catalysts contributes largely to the outstanding OER activity, and thicker reconstructed surface layer results in more active sites of catalysts [54]. However, combined with our experimental results, we can deduce that the final OER activity is correlated with the bulk chemistry of the reconstructed surface and not the degree of surface rearrangement. Therefore, this observation calls for a more detailed study of the surface chemical properties of the pristine perovskite materials. In general, Fe (*x* = 0.1) doping can not only improve the reconstruction rate of LaNiO_3_ but also decrease the activation degree and reconstruction degree with LaNi_0.9_Fe_0.1_O_3_/Ni_0.9_Fe_0.1_OOH showing the most stable interface structure for further catalysis.

**Fig. 6:**
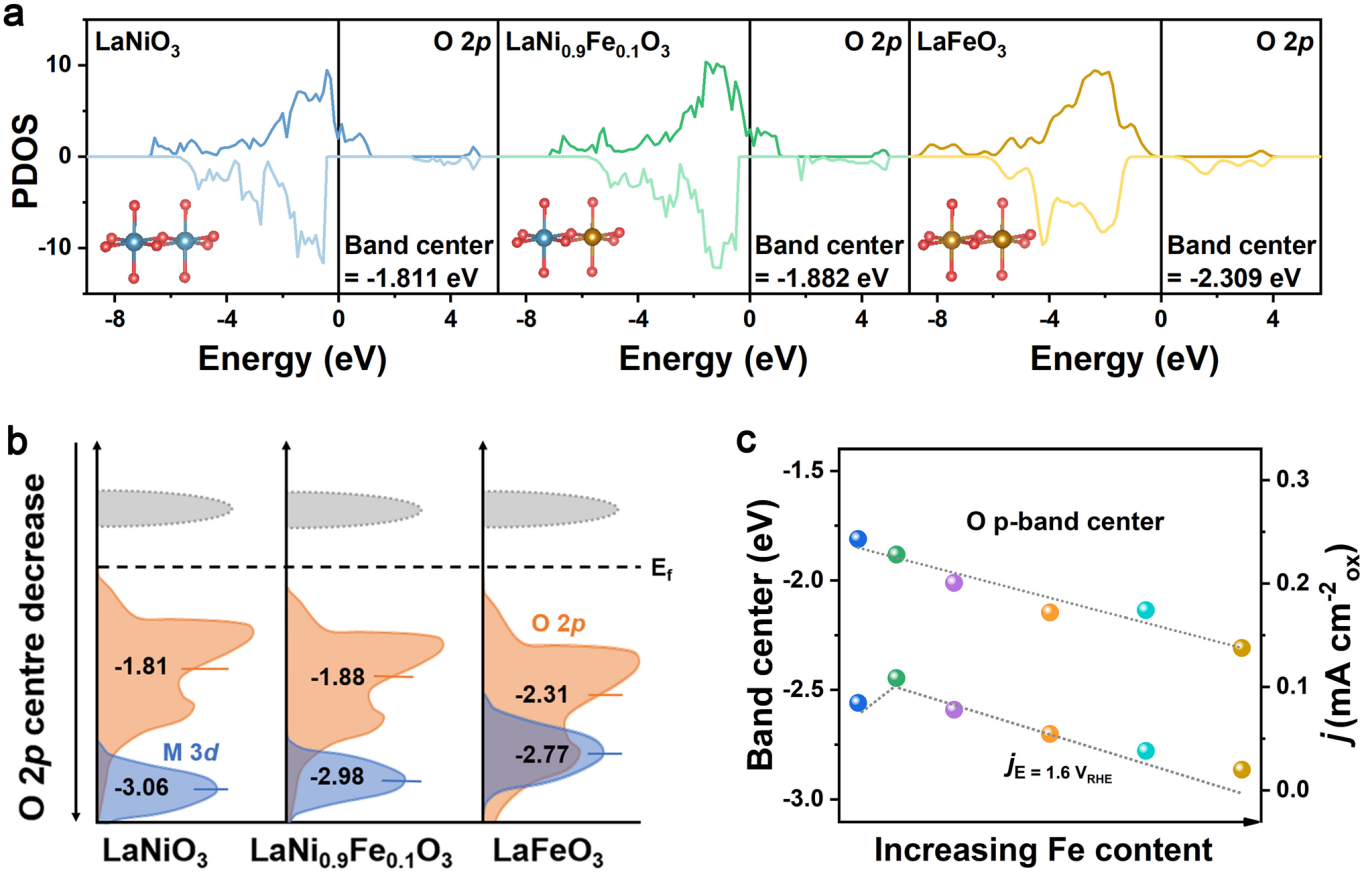
**a** Computed M 3*d* and O 2*p* PDOS of LaNiO_3_, LaNi_0.9_Fe_0.1_O_3_, and LaNi_0.75_Fe_0.25_O_3_. **b** Schematic band diagrams of LaNiO_3_, LaNi_0.9_Fe_0.1_O_3_ and LaFeO_3_. **c** Current densities at 1.6 V and the O 2*p* band center relative to the Fermi level for LaNi_1-x_Fe_x_O_3_

As mentioned previously, Fe doping shows a direct effect on both OER activity and thickness of amorphous reconstructed surface layer. Hence, we performed DFT calculations to further investigate the mechanism of OER activity enhancement and surface reconstruction. The projected density of state (PDOS) of LaNiO_3_, LaNi_0.9_Fe_0.1_O_3_, and LaFeO_3_ and their band center energies are presented in Fig. 6a. The incorporation of Fe into the perovskite is projected to shift the O 2*p* band center toward lower energy, farther away from the Fermi level (Fig. 6b). The PDOS results show that substitution of 10% Ni by Fe can reduce the O 2*p* level of LaNiO_3_ from −1.811 to −1.882 eV. Earlier studies have revealed that uplifting the O 2*p* band level toward the Fermi level can result in a more dominant lattice oxygen oxidation mechanism for oxides. However, the oxidation of lattice oxygen is always featured with great surface reconstruction and thus inevitable structural instability [18]. Thus, due to the lowered O 2*p* band center, the lattice oxygen oxidation process is suppressed, resulting in enhanced structural stability of the surface of the Fe-doped perovskite. Moreover, the position of the O 2*p* band center shows a trend consistent with the activation degree (Fig. 2f) of LaNi_1-x_Fe_x_O_3_, but inconsistent with their OER activity (Fig. 6c). We thus believe that the OER activity of LaNi_1-x_Fe_x_O_3_ oxides is dominated by the surface structure and composition after self-rearrangement, while their surface reconstruction degree is governed by the predicted O 2*p* level.

## Conclusions

In summary, with LaNi_1-x_Fe_x_O_3_ perovskite oxides as research model, we found that Fe substitution can manipulate the intrinsic activity, activation degree, reconstruction degree, and reconstruction rate. Among these substituted oxides, LaNi_0.9_Fe_0.1_O_3_ after surface reconstruction exhibits the best electrocatalytic performance. The volcano-shaped activity trend and the thinning of reconstructed surface with the increase of Fe doping demonstrate that OER activity mainly depends on the composition of the LaNi_1-x_Fe_x_O_3_ perovskite, while not the surface reconstruction degree. The reconstructed amorphous layer composed of highly active Ni_0.9_Fe_0.1_OOH on LaNi_0.9_Fe_0.1_O_3_ with balanced reconstruction degree and reconstruction rate shows outstanding intrinsic catalytic activity and long-term stability. By combining experimental measurements and theoretical calculations, we found that Fe substitution in LaNiO_3_ perovskite oxide can facilitate Ni^3+^ to reach dynamic equilibrium, leading to a stable active reconstructed layer. The downshift of O 2*p* band center by Fe incorporation suppresses the oxidation of lattice oxygen under OER conditions and thus provides stable surface chemistry to contribute excellent stability. The findings here illustrate the importance of surface composition after reconstruction, which is more significant in determining the activity than the degree of surface reconstruction.

## Supplementary Information

Below is the link to the electronic supplementary material.

Supplementary file1 (DOCX 19133 KB)
