# Supplementary material for: Deciphering Water Oxidation Catalysts: The Dominant Role of Surface Chemistry over Reconstruction Degree in Activity Promotion
Source: Nanomicro Lett. 2024 Nov 26;17:70. doi: 10.1007/s40820-024-01562-7 (PMC11599692; doi:10.1007/s40820-024-01562-7)
Supplement: Supplementary file 1 — Supplementary file1 (DOCX 19133 KB) [file 40820_2024_1562_MOESM1_ESM.docx]

Supporting Information for

**Deciphering Water Oxidation Catalysts: The Dominant Role of Surface Chemistry over Reconstruction Degree in Activity Promotion**

Li An^1,#^, Jianyi Li^1,#^, Yuanmiao Sun^2,3,#^, Jiamin Zhu^1,#^, Justin Zhu Yeow Seow^2^, Hong Zhang^2^, Nan Zhang^1^, Pinxian Xi^1,4,^ *, Zhichuan J. Xu^2,^ *, and Chun-Hua Yan^1,5^

^1^State Key Laboratory of Applied Organic Chemistry, Key Laboratory of Nonferrous Metal Chemistry and Resources Utilization of Gansu Province, Frontiers Science Center for Rare Isotopes, College of Chemistry and Chemical Engineering, Lanzhou University, Lanzhou, 730000, P. R. China

^2^School of Materials Science and Engineering, Nanyang Technological University, Singapore

^3^Faculty of Materials Science and Energy Engineering, Institute of Technology for Carbon Neutrality, Shenzhen Institute of Advanced Technology, Chinese Academy of Sciences, Shenzhen 518055, P. R. China

^4^Key Laboratory of Electromagnetic Materials and Devices, National Center for International Research on Photoelectric and Energy Materials, School of Materials and Energy, Electron Microscopy Center, Yunnan University, Kunming 650091, P. R. China

^5^ State Key Laboratory of Baiyunobo Rare Earth Resource Researches and Comprehensive Utilization, Baotou Research Institute of Rare Earths, Baotou 014030, P. R. China

^6^Beijing National Laboratory for Molecular Sciences, State Key Laboratory of Rare Earth Materials Chemistry and Applications, PKU-HKU Joint Laboratory in Rare Earth Materials and Bioinorganic Chemistry, Peking University, Beijing 100871, P. R. China

^#^ Li An, Jianyi Li, Yuanmiao Sun, and Jiamin Zhu contributed equally to this work.

*Corresponding authors. E-mail: [xipx@lzu.edu.cn](mailto:xipx@lzu.edu.cn) (Pinxian Xi), [xuzc@ntu.edu.sg](mailto:xuzc@ntu.edu.sg) (Zhichuan J. Xu)

**S1 Experimental Section**

**S1.1 Structural characterizations**

Powder X-ray diffraction (XRD) patterns were collected on a Rigaku D/Max-2400 diffractometer with Cu-Kα radiation (λ = 1.54178 Å) and 2θ values from 10^o^ to 90^o^ under a constant voltage of 40 kV. The morphology of the products was analyzed by field emission scanning electron microscope (FE-SEM, FEI Sirion-200 SEM) at an acceleration voltage of 5 kV. All samples were coated with a thin layer of gold before FE-SEM observations. The transmission electron microscopy (TEM) and high-resolution transmission electron microscopy (HRTEM) images were collected on Tecnai G2 F30 Field Emission Transmission Electron Microscope. Atomic-scale STEM images were recorded on a probe aberration-corrected STEM (Cubed Titan G2 60-300, FEI, USA) operated at 300 kV. X-ray photoelectron spectroscopy (XPS) was carried out on a VG ESCALAB 220I-XL device with Mg Kα (*hv* = 1253.6 eV) excitation source. The binding energies obtained in the XPS spectral analysis were corrected for specimen charging by referencing C 1s to 284.8 eV. The standard deviation for the binding energy (BE) values was 0.1 eV. N_2_ adsorption/desorption measurement was carried out using an Autosrob-1 (Quantachrome INSTRUMENTS). Inductively coupled plasma optical emission spectrometry (ICP-OES) analyses were performed on a Plasma Quant PQ9000 ICP spectrometer. The X-ray absorption fine structure spectra (at the K-edges of Ni and Fe) analyses were performed at the 1W1B station in Beijing Synchrotron Radiation Facility (BSRF) using a Si (111) double-crystal monochromator. The storage rings of BSRF were operated at 2.5 GeV with a maximum current of 250 mA. The data reduction and data analysis were performed with the Athena, Artemis, and IFEFFIT software packages.

In-situ Raman spectroscopy was performed using the in-situ electrochemical Raman cell (031-2H) with a saturated Ag/AgCl reference electrode and a Pt ring counter electrode. Potential-dependent in-situ Raman spectra tests were carried out in 1.0 M KOH as soon as a stable chronoamperometric curve was obtained to detect the surface chemical composition and structural evolution of materials. In-situ attenuated total reflection infrared (ATR-IR) spectra were obtained using a customized cell, with a saturated Ag/AgCl reference electrode and a Pt ring counter electrode in 1.0 M KOH to observe the surface-adsorbed species. Differential electrochemical mass spectroscopy (DEMS) measurements were carried out to determine the ^18^O-labeled volatile reaction products using a QAS 100 device (Linglu Instruments, Shanghai) in ^16^O KOH solution. For DEMS measurements, the working electrodes were made by sputtering Au onto a 50 μm thick-porous polytetrafluoroethylene films and then dropping the catalysts onto Au with a loading mass of 1 mg cm^-2^. In this time, the saturated Ag/AgCl electrode and Pt wire were used as the reference electrode and counter electrode, respectively. To label the catalysts by ^18^O isotopes, we set 10 CV cycles n ^18^O-labeled 1 M KOH solution with the voltage window range of 0.2-0.8 V (Ag/AgCl) at a scan rate of 10 mV s^-1^. After this step, this ^18^O-labeled catalyst was thoroughly rinsed by ^16^O water (more than five times) to remove the remaining ^18^O species adsorbed on the surface, thus avoid disturbing observed ^34^O_2_ (^16^O^18^O) signals. Finally, the ^18^O-labeled electrode was performed by CV cycles in in ^16^O 1.0 M KOH solution with the same set parameters and the generated gas products during this process were measured by mass spectroscopy in real time.

**Note S1** **X-ray absorption spectroscopy data analysis**

Data merging, normalization, and fitting of the XANES and EXAFS spectra were performed using the Demeter software package. The data reduction and data analysis were performed with the Athena, Artemis, and IFEFFIT software packages. The k^3^-weighted Fourier transforms for all the EXAFS data were conducted in the k-range of 2-12 Å^-1^. EXAFS were fitted using the normal spinel LaNiO_3_ structure as the starting structure model. The R-ranges for fitting all the EXAFS data were set as 1.0 - 2.0 Å. The edge position was accurately estimated by an integral method in the range of 0.15~1.0 normalized *χμ* (E), where *μ* is the normalized absorbance.

**Note S2 “Fe-free” alkaline solution preparation**

The electrolytes used for the electrochemical measurements are prepared by semiconductor grade potassium hydroxide (KOH) pellets (Sigma-Aldrich) with a purity of 99.999%. To avoid Fe contamination, all electrochemical characterizations were conducted in plastic cells.

**Note S3** **TOF estimation methodology**

The TOF is calculated from the equation: TOF = *jA*/4*Fn*

where *j* is the specific-surface-area-normalized current density (mA cm^-2^_ox_) obtained at an overpotential (η) = 300 mV vs. RHE; *A* is the specific surface area of oxide measured by BET in cm^2^_ox_; The factor 4 represents 4 electron transfer involved in evolving a mole of O_2_; *F* is Faraday’s constant (96485.3 C mol^-1^); and *n* is the number of moles of active sites on the electrode.

*n* is estimated from the integration of Ni reduction peak at 1.35 V vs. RHE (assuming a one-electron process of Ni^4+^/Ni^3+^) using the following formula: *n*_redox_ = Q/F

where Q of the reduction peak is obtained by dividing the area under the relevant peak in the CV by scan rate, and F is the Faraday’s constant. The TOF obtained using this method is denoted as TOF_redox_ [43]. It should be noted that the contribution of Fe active sites in TOF_redox_ estimation is deemed negligible, which is consistent with the cited reports [S1-S3].

**Note S4 Calculation of redox charge**

The difference in the electrochemically active metal site density on the surface was examined by analyzing the anodic oxidation charge (*q*_a_) and cathodic oxidation charge (*q*_c_) measured in 1.0 M KOH electrolyte, and the redox charge in CV plots is defined before the OER region [S4, S5]. The redox charge can be used as a proxy of the number of surface-active sites.

**S2 Supplementary Figures and Tables**


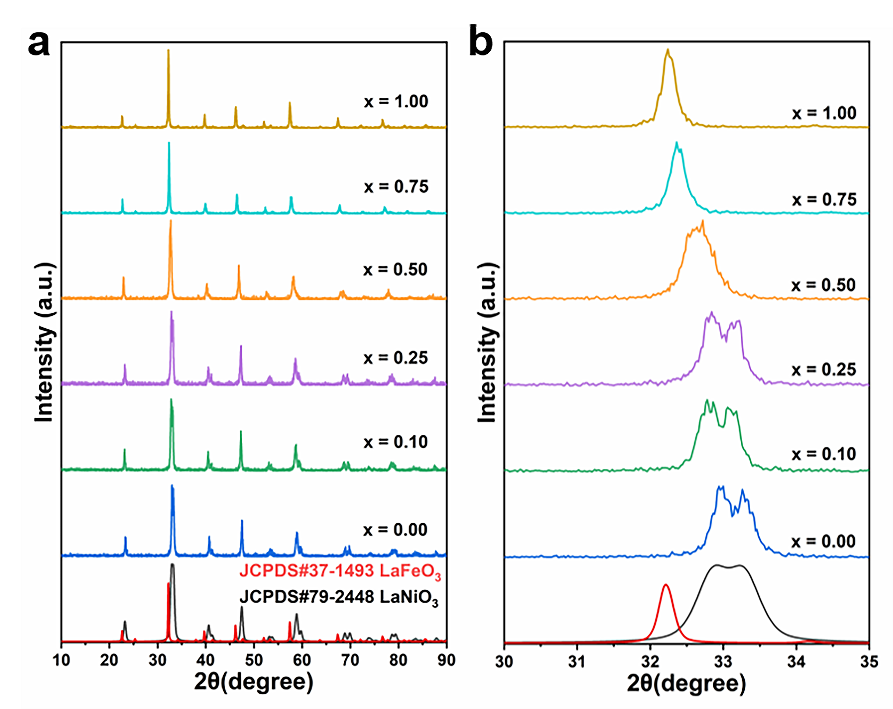


**Fig. S1** Powder XRD patterns of as-synthesized LaNi_1-x_Fe_x_O_3_ (x = 0.00, 0.10, 0.25, 0.50, 0.75, 1.00) perovskite oxides.

The XRD patterns of the as-prepared LaNiO_3_, LaNi_0.9_Fe_0.1_O_3,_ and LaNi_0.75_Fe_0.25_O_3_ correspond to standard rhombohedral perovskite structure with R-3c space group (JCPDS: 79-2448), while those of LaNi_0.25_Fe_0.75_O_3_ and LaFeO_3_ correspond to orthorhombic phase structure with Pn*a space group (JCPDS: 37-1493).


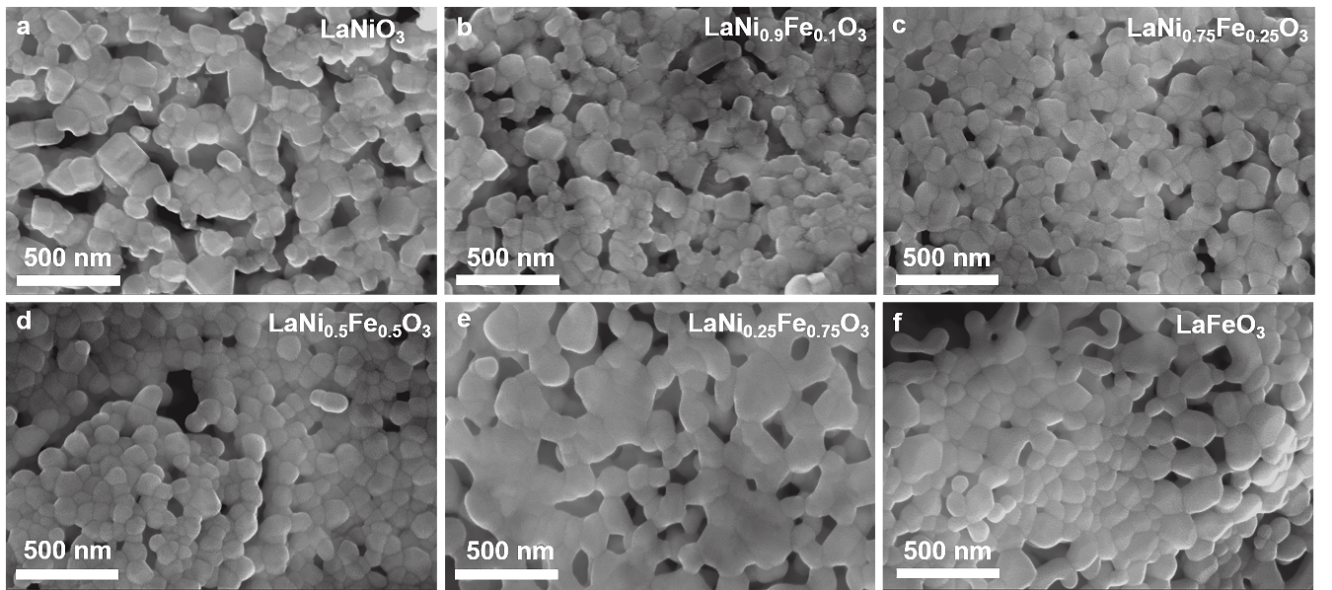


**Fig. S2** SEM images of LaNi_1-x_Fe_x_O_3_ (x = 0.00, 0.10, 0.25, 0.50, 0.75, 1.00) perovskite series.


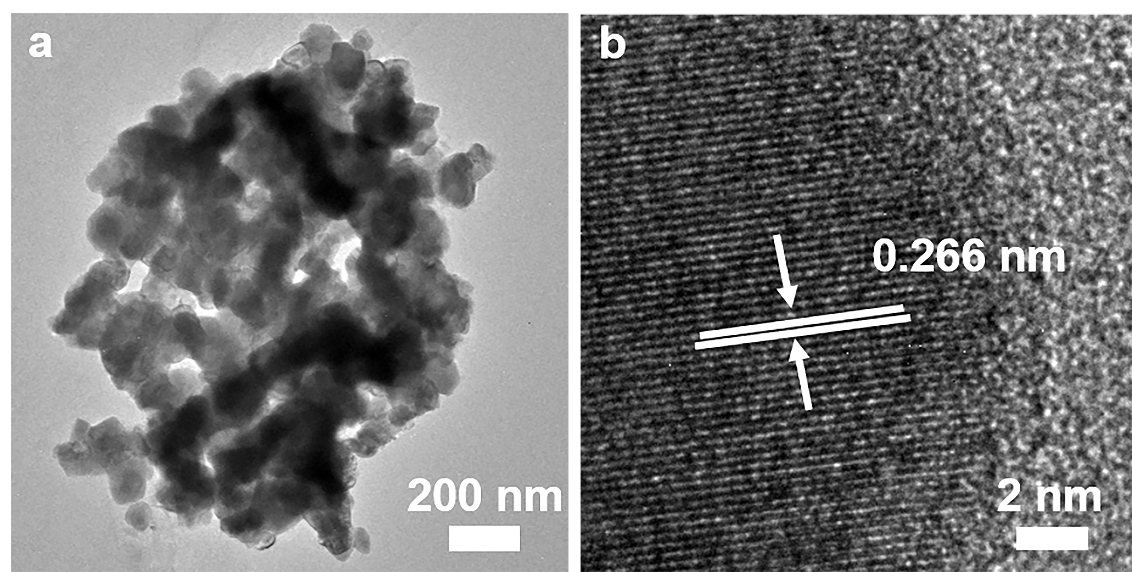


**Fig. S3** TEM and HRTEM images of LaNi_0.9_Fe_0.1_O_3_ perovskite.


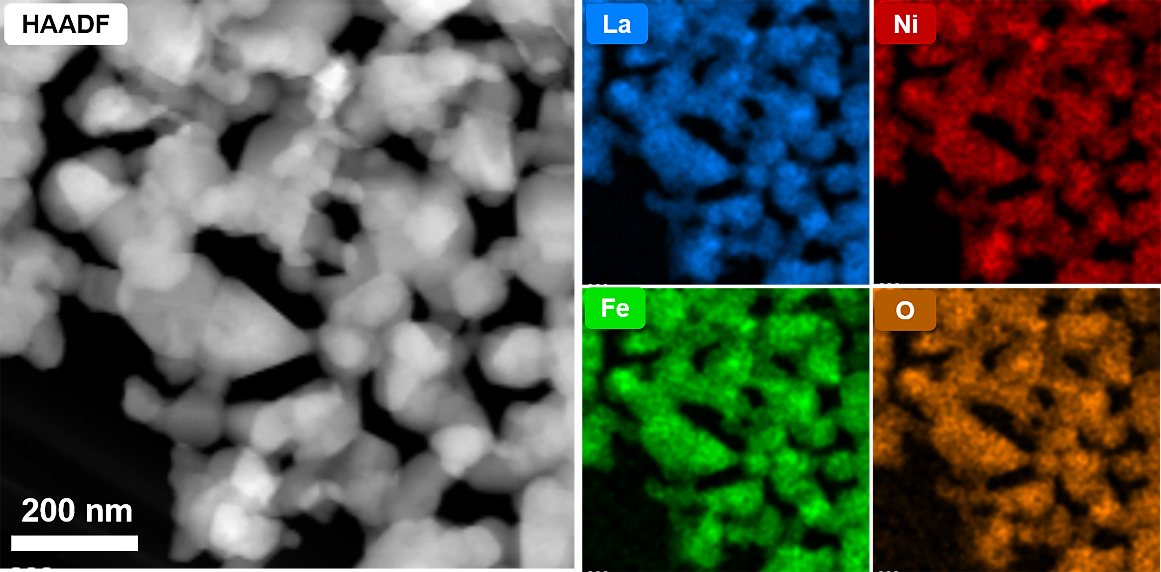


**Fig. S4** EDS elemental mapping images of the as-synthesized LaNi_0.9_Fe_0.1_O_3_ perovskite oxide.


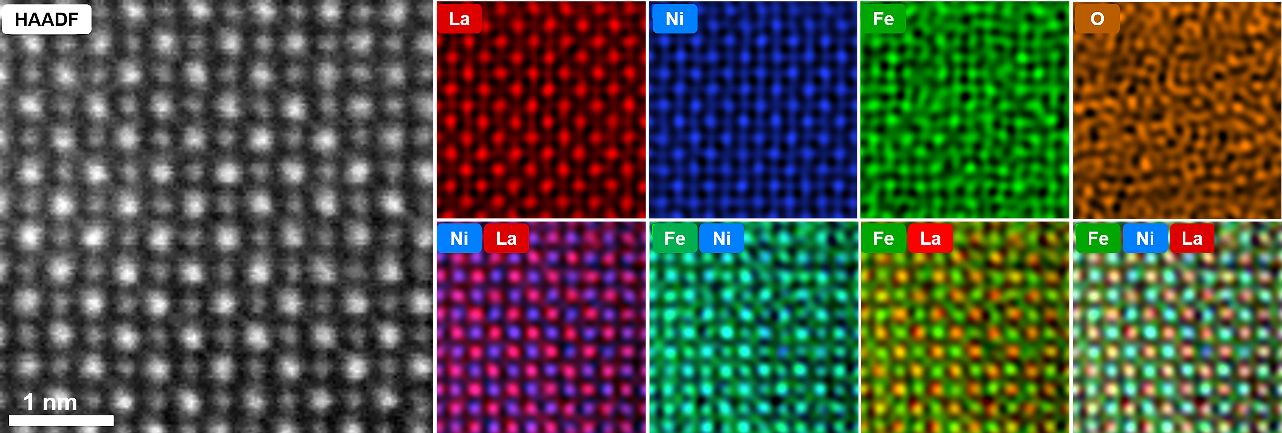


**Fig. S5** HAADF-STEM image and corresponding EDS atomic elemental mappings of the as-synthesized LaNi_0.9_Fe_0.1_O_3_ perovskite oxide


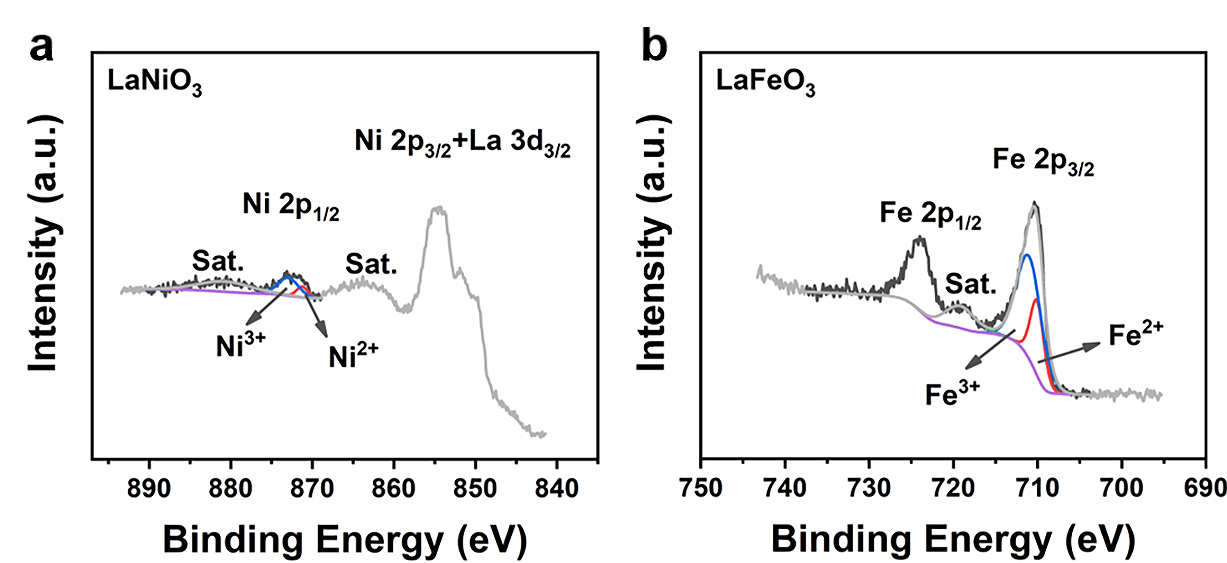


**Fig. S6** XPS core-level spectra of (**a**) Ni 2p of LaNiO_3_ and (**b**) Fe 2p of LaFeO_3_ with peak fitting results.

Figure S6a shows the peaks at 871.2 and 873.0 eV, corresponding to the Ni^2+^ 2p_1/2_ and Ni^3+^ 2p_1/2_ respectively, indicating the coexistence of Ni^2+^ and Ni^3+^ on LaNiO_3_. Besides, Fig. S6b shows the main peaks at 710.1 and 711.0 eV attributed to Fe^2+^ 2p_3/2_ and Fe^3+^ 2p_3/2_ respectively, demonstrating the coexistence of Fe^2+^ and Fe^3+^ on LaFeO_3_.


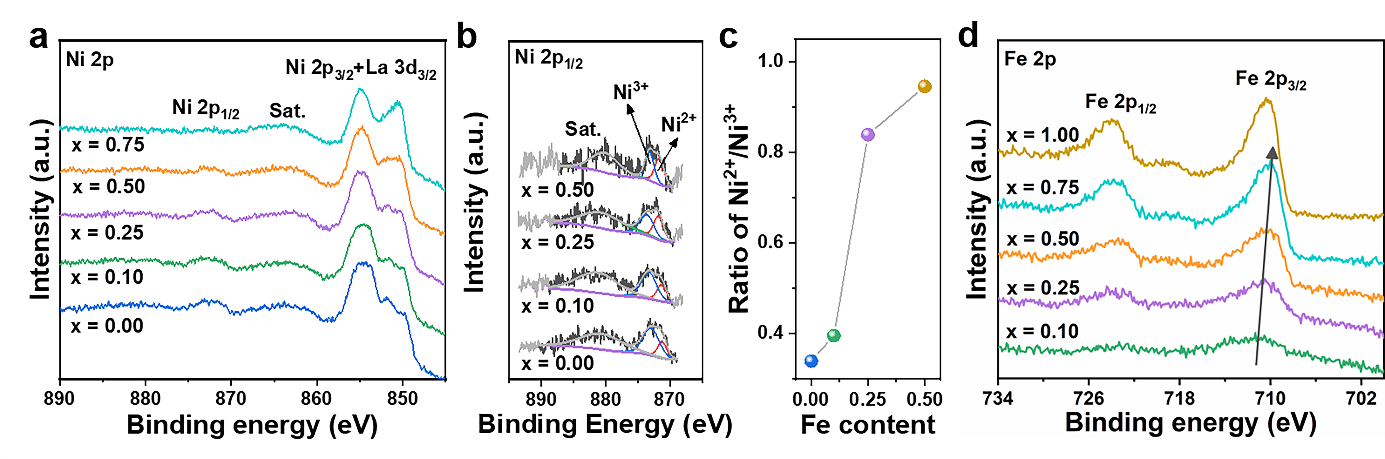


**Fig. S7** XPS core-level spectra of (**a**) Ni 2p and (**b**) Ni 2p_1/2_ of LaNi_1-x_Fe_x_O_3_, and (**c**) ratio of Ni^2+^/Ni^3+^ estimated from XPS spectra in (**b**). (**d**) XPS core-level spectra of Fe 2p of LaNi_1-x_Fe_x_O_3_.

Owing to the high overlap of Ni 2p_3/2_ and La 3d_3/2_ peaks [S6, S7], it is difficult to reliably estimate the Ni^2+^/Ni^3+^ ratio, and hence, we analyzed the Ni^2+^/Ni^3+^ ratio from Ni 2p_1/2_. As shown in Fig. S7b and S7c, the Ni^2+^/Ni^3+^ ratio increases evidently with Fe content for 0 ≤ x ≤0.5. While, for LaNi_0.25_Fe_0.75_O_3_, the signal-to-noise ratio of Ni 2p_1/2_ peaks is too low for reliable fitting owing to the low content of Ni. Nonetheless, based on the X-ray absorption near edge structure spectra in Fig. 1C (Main Text), it has been demonstrated that the ratio of Ni^2+^/Ni^3+^ increases with increasing Fe content in LaNi_1-x_Fe_x_O_3_ (x = 0.00, 0.10, 0.25, 0.50, 0.75).

In addition, the high-resolution Fe 2p_3/2_ XPS spectrum of LaFeO_3_ shows a slight positive shift compared to that of LaNi_0.25_Fe_0.75_O_3_. This abnormal phenomenon can be explained by the relatively stable and ordered surface structure of LaFeO_3_ with very little oxygen vacancies owing to the stable valence of ferric iron. Meanwhile, the valence state of Fe decreases with the increase of Fe content (Fig. 7D), demonstrating a strong electronic interaction between Ni and Fe.


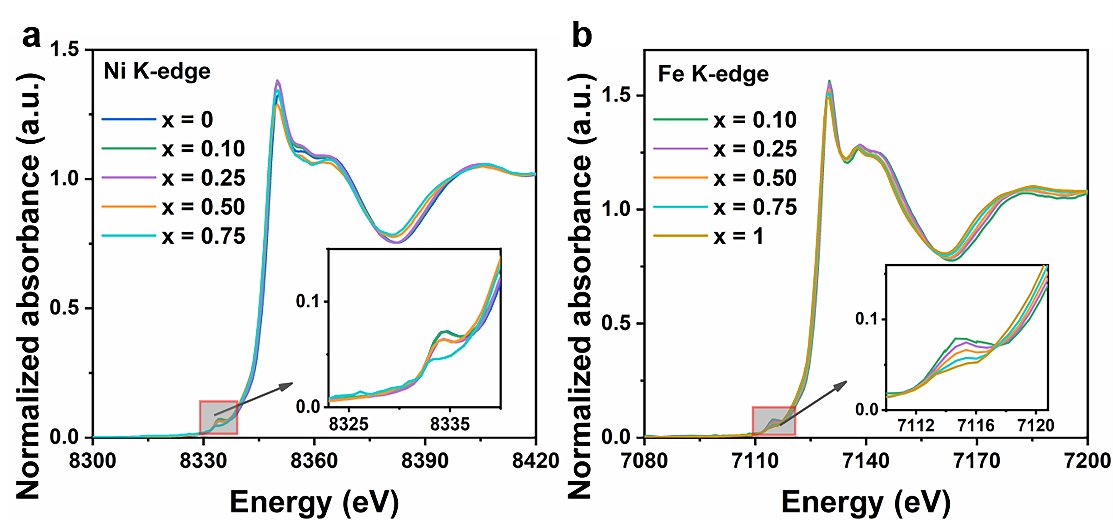


**Fig. S8** Normalized (**a**) Ni K-edge XANES spectra and (**b**) Fe K-edge XANES spectra of LaNi_1-x_Fe_x_O_3_. Corresponding insets show the pre-edge features of Co K-edge XANES spectra.

**
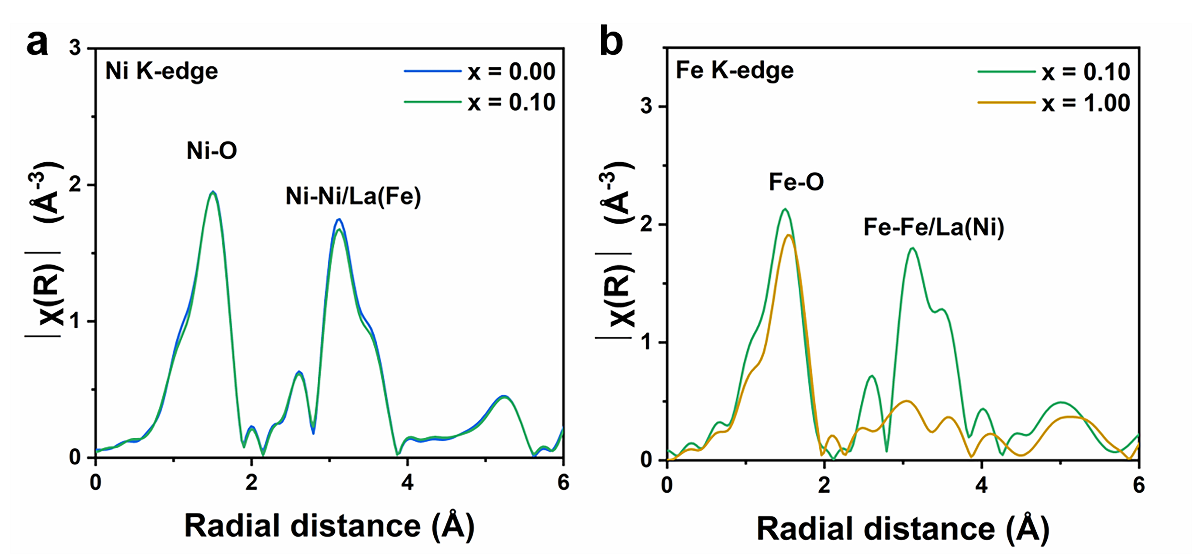
**

**Fig. S9** (**a**) FT *k_3_χ(R)* Ni K-edge EXAFS of LaNiO_3_ and LaNi_0.9_Fe_0.1_O_3_ in R space. (**b**) FT *k_3_χ(R)* Fe K-edge EXAFS of LaFeO_3_ and LaNi_0.9_Fe_0.1_O_3_ in R space.

Fourier transform (FT) extended X-ray absorption fine structure (EXAFS) can be used to reveal the local geometrical structures and local chemical environment of Ni and Fe. Two major peaks were observed in the EXAFS spectra of Ni and Fe, representing the neighboring atomic shells in the vicinity of atoms Ni and Fe, respectively. The first shell around 1.5 Å is attributed to Ni-O and Fe-O coordination, while the second shell located around 3.5 Å corresponds to the Ni-Ni/La(Fe) and Fe-Fe/La(Ni) coordination, respectively. The changed Ni-Ni/La(Fe) and Fe-Fe/La(Ni) bonds can be attributed to the difference in surface atom arrangement and octahedral distortion. Besides, compared with LaFeO_3_, the coordination number of LaNi_0.9_Fe_0.1_O_3_ is greatly increased, illustrating that the valence state of Fe is changed by Ni and demonstrating the strong interaction between metal Ni and Fe [S6, S7].

**
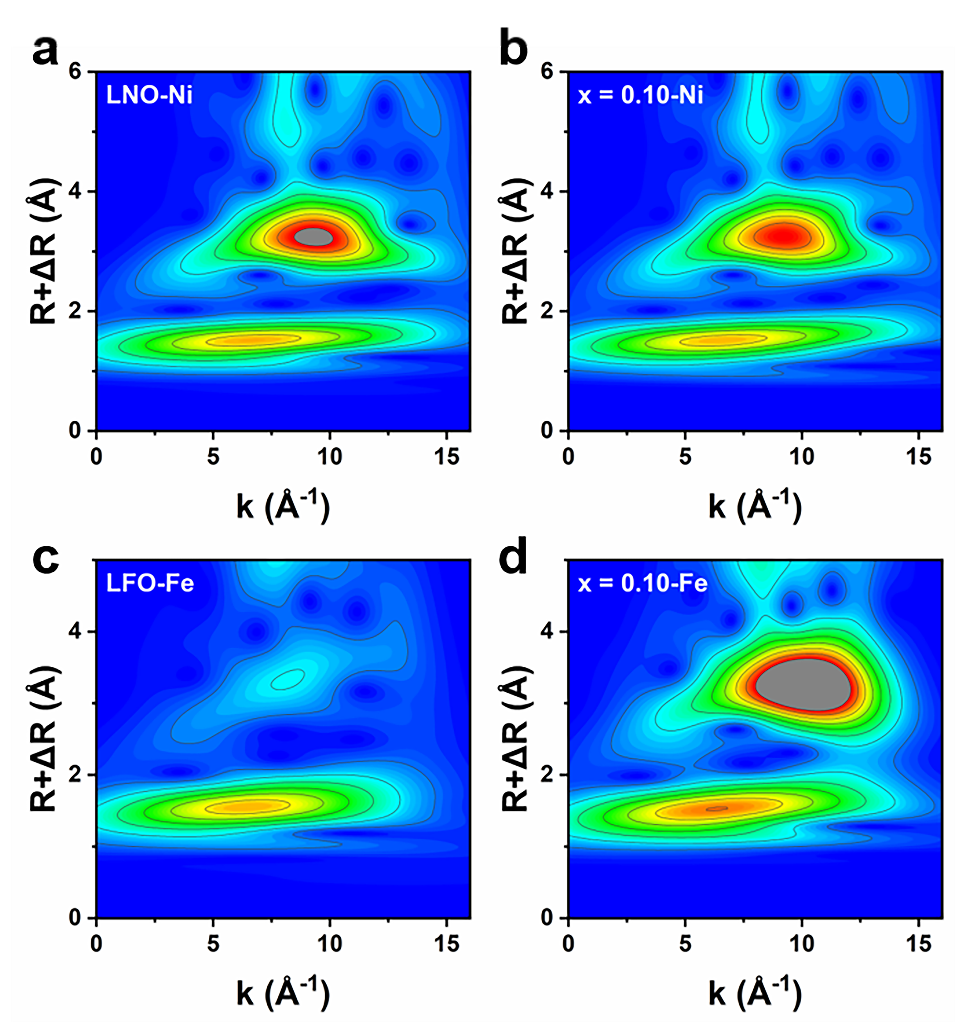
**

**Fig. S10** WT analysis of the Ni K-edge EXAFS spectra of (**a**) LaNiO_3_ and (**b**) LaNi_0.9_Fe_0.1_O_3_, and the Fe K-edge EXAFS spectra of (**c**) LaFeO_3_ and (**d**) LaNi_0.9_Fe_0.1_O_3_.

The wavelet transformation (WT) is a powerful tool to identify the contribution of different scattering paths and investigate the character of the neighboring atoms around the metal center [S8, S9]. In this way, WT analysis was further applied and two main maxima can be identified in all of these samples: the one located at k ≈ 7 Å^-1^ (R ≈ 1.5 Å) corresponds to the M-O scattering path in the first shell, and the other one at k ≈ 10 Å^-1^ (R ≈ 3 .5 Å) is assigned to M-M path in the second shell. In detail, the maximum associated with the Ni-M scattering path decreases while the maximum associated with the Fe-M scattering path becomes more obvious, demonstrating the interaction of Ni host and Fe dopant in the given perovskite structure.

**
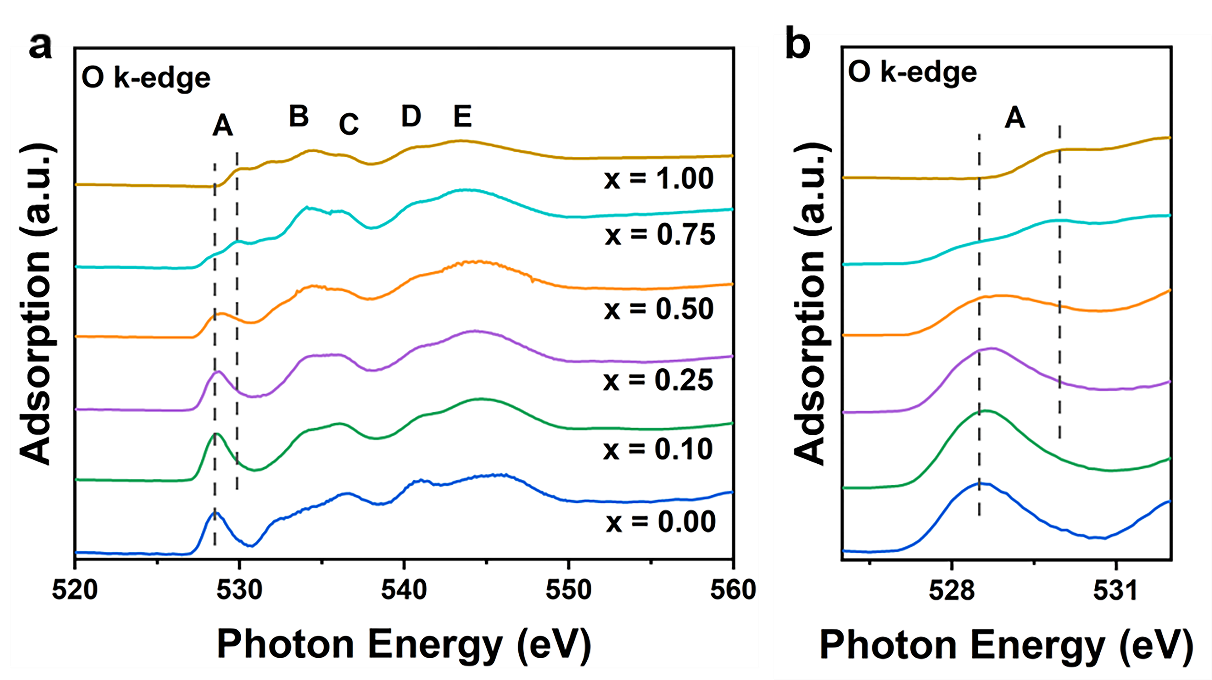
**

**Fig. S11** (**a**) O K-edge XAS spectra and (**b**) the corresponding magnified XAS spectra of peak A for LaNi_1-x_Fe_x_O_3_ (x = 0.00, 0.10, 0.25, 0.50, 0.75, 1.00) perovskite oxides.

XAS (X-ray absorption spectroscopy) is a widely accepted detection tool to study the electronic structure of materials. Five main peaks were detected in the O K-edge XAS. Peak A corresponds to the transition from the core level to the hybridization states O 2p and M (Ni/Fe) 3d; peaks B and C represent the hybridization between O 2p and La 5d; peaks D and E can be considered as representations of the Co 4sp-O 2p hybrid states [S10].

**
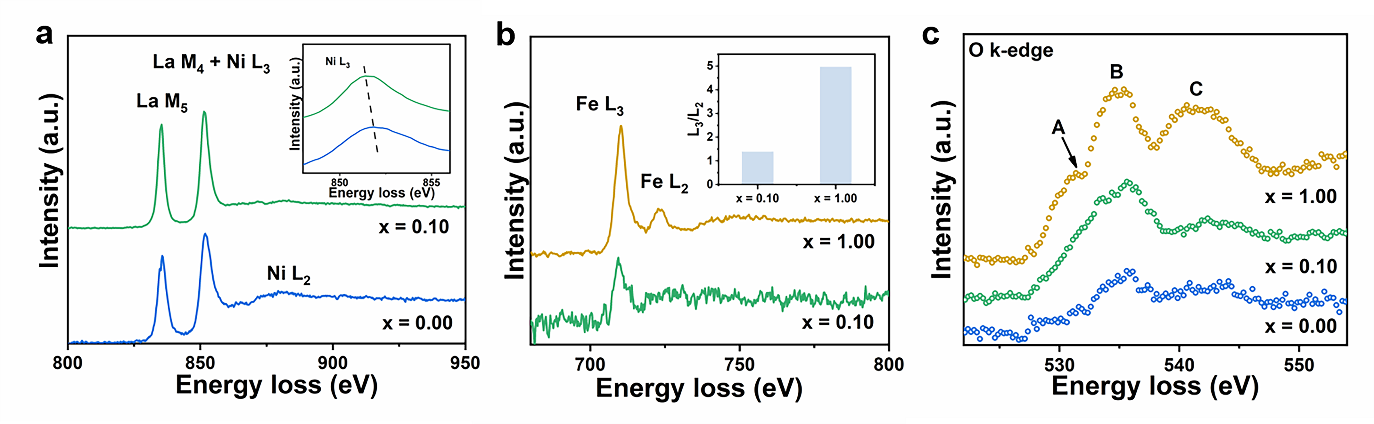
**

**Fig. S12** EELS spectra of (**a**) Ni L-edge for LaNiO_3_ and LaNi_0.9_Fe_0.1_O_3_, (**b**) Fe L-edge for LaFeO_3_ and LaNi_0.9_Fe_0.1_O_3_, and (**c**) O K-edge for LaNiO_3_, LaFeO_3_, and LaNi_0.9_Fe_0.1_O_3_.

The L_3_/L_2_ ratios of Fe were calculated by the Hartree–Slater cross-section method [S11]. Lower L_3_/L_2_ corresponds to higher oxidation states of the Fe cations. For the O K-edge spectra, three characteristic peaks labeled as A, B, and C near the edge onset can be observed, which were assigned to the hybridization of O 2p with M 3d, La 5d, and M 4sp orbitals, respectively. The disappearance of pre-peak A can be generally attributed to oxygen vacancy formation due to the change in Ni and Fe oxidation states.

**
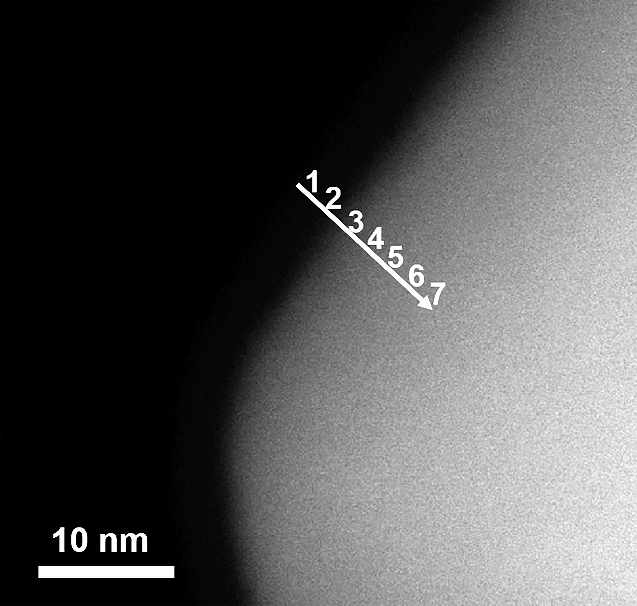
**

**Fig. S13** STEM image of as-synthesized LaNi_0.9_Fe_0.1_O_3_ with the scanning pathway from Point 1 to Point 7.

**
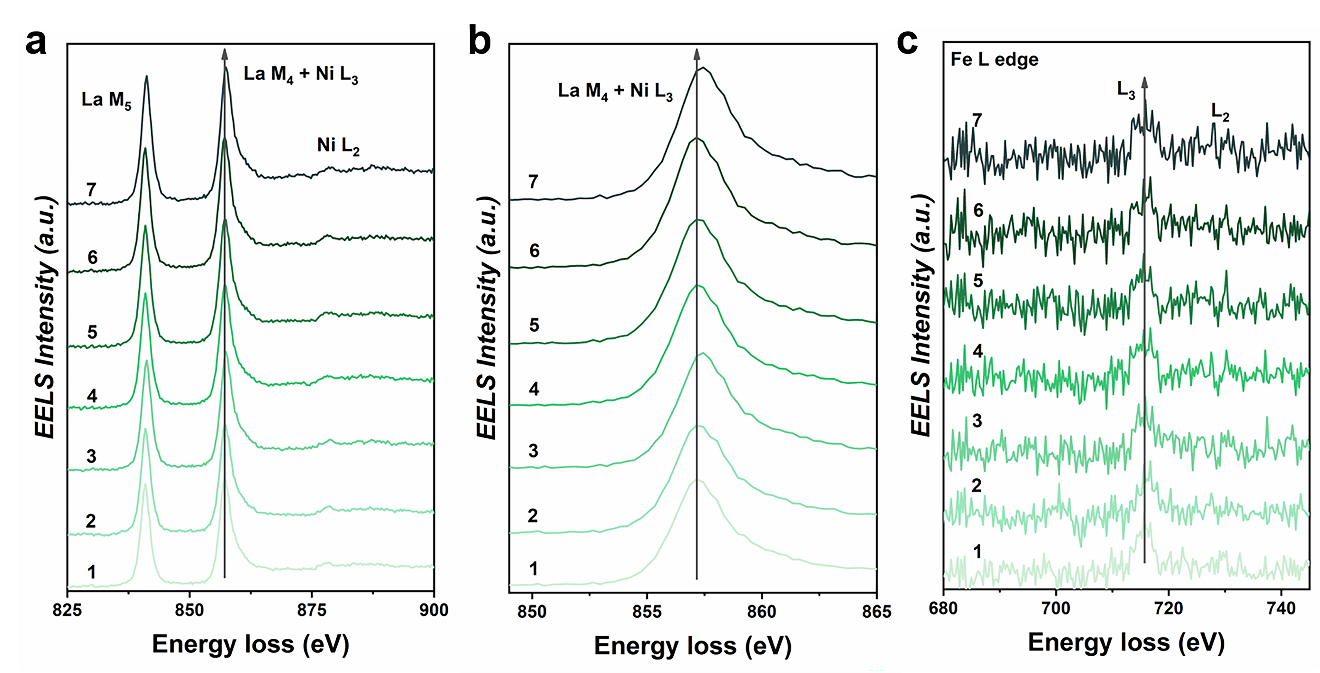
**

**Fig. S14** EELS spectra of (**a, b**) Ni L-edge and (**c**) Fe L-edge for the as-synthesized LaNi_0.9_Fe_0.1_O_3_ along the scanning pathway shown in Fig. S13 to detect the charge distributions of metal cations [S12].

**

**

**Fig. S15** The chronopotentiometry (CP) test of LaNi_0.9_Fe_0.1_O_3_ perovskite oxide loaded on carbon paper in 1.0 M KOH at a constant current density of 10 mA cm^-2^_disk_ for 100 h. The robust structure and good stability are attributed to the stable bulk structure of the perovskite oxide despite the occurrence of surface reconstruction.

**
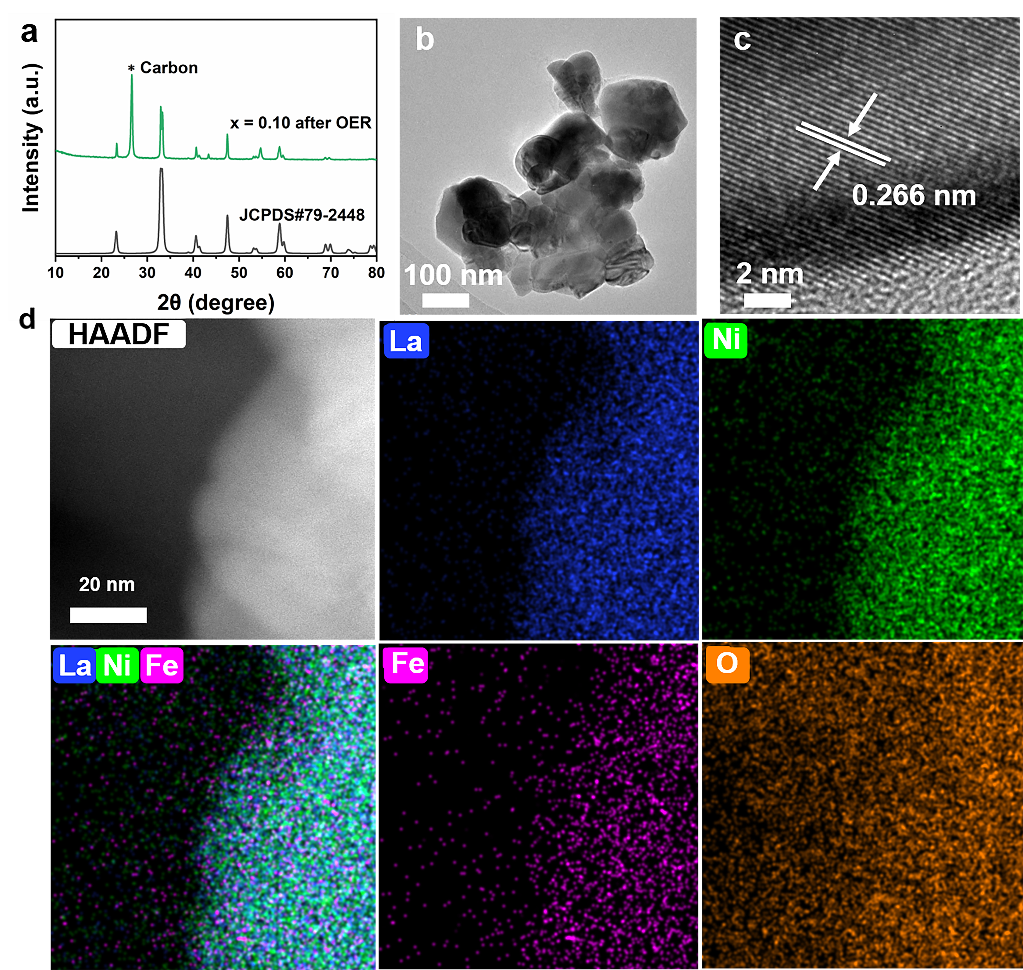
**

**Fig. S16** (**a**) XRD pattern, (**b**) TEM image, (**c**) HRTEM, and (**d**) corresponding EDS mappings of LaNi_0.9_Fe_0.1_O_3_ perovskite oxide after long-term chronopotentiometry test. The peaks at ~ 43^o^ and 55^o^ can be attributed to the newly formed NiFeOOH at the surface [S13].

**
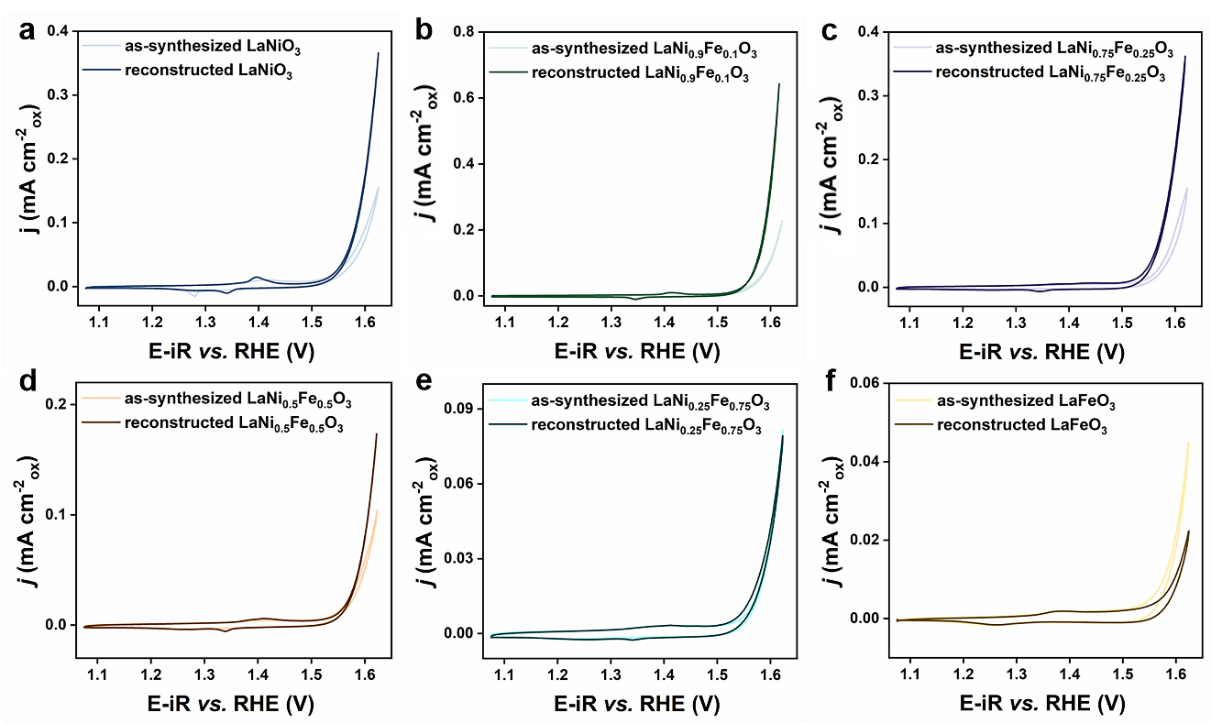
**

**Fig. S17** Evolutive CV of LaNi_1-x_Fe_x_O_3_ (x = 0.00, 0.10, 0.25, 0.50, 0.75 and 1.00) before and after reconstruction process in 1.0 M KOH at 10 mV s^-1^ between 1.075 and 1.625 V (vs. RHE).

Electrochemical activation process was performed on LaNi_1-x_Fe_x_O_3_ at a scan rate of 100 mV s^-1^, while the OER activity profiles were obtained at a scan rate of 10 mV s^-1^. To avoid the possibility of Fe-impurity-induced activity enhancement, Fe-free electrolyte prepared from electronic grade KOH (99.999% purity) was used for the OER characterizations, detailed procedures of which are provided in Note S2.

**
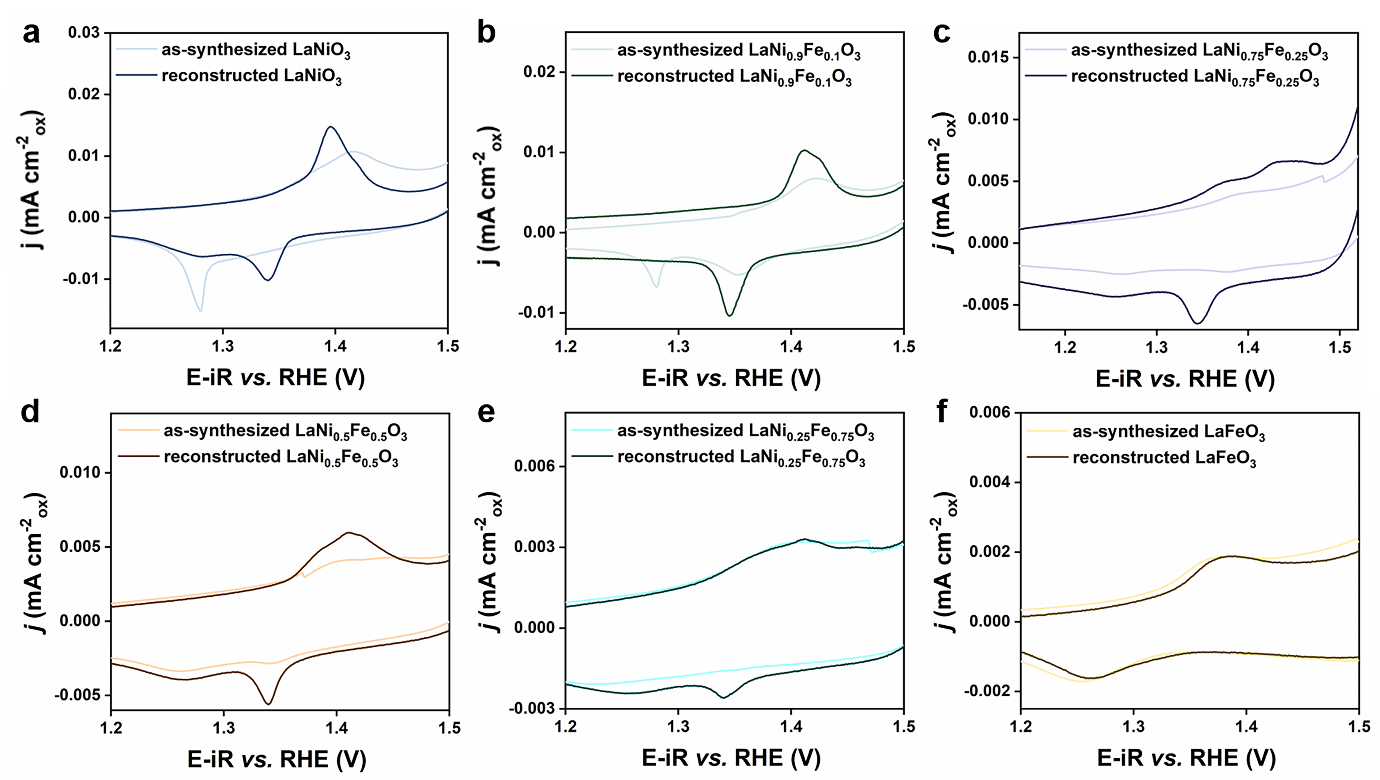
**

**Fig. S18** Magnified CV showing the pre-OER redox peaks features from 1.2 to 1.5 V (vs. RHE) for LaNi_1-x_Fe_x_O_3_ (x = 0.00, 0.10, 0.25, 0.50, 0.75 and 1.00).

**
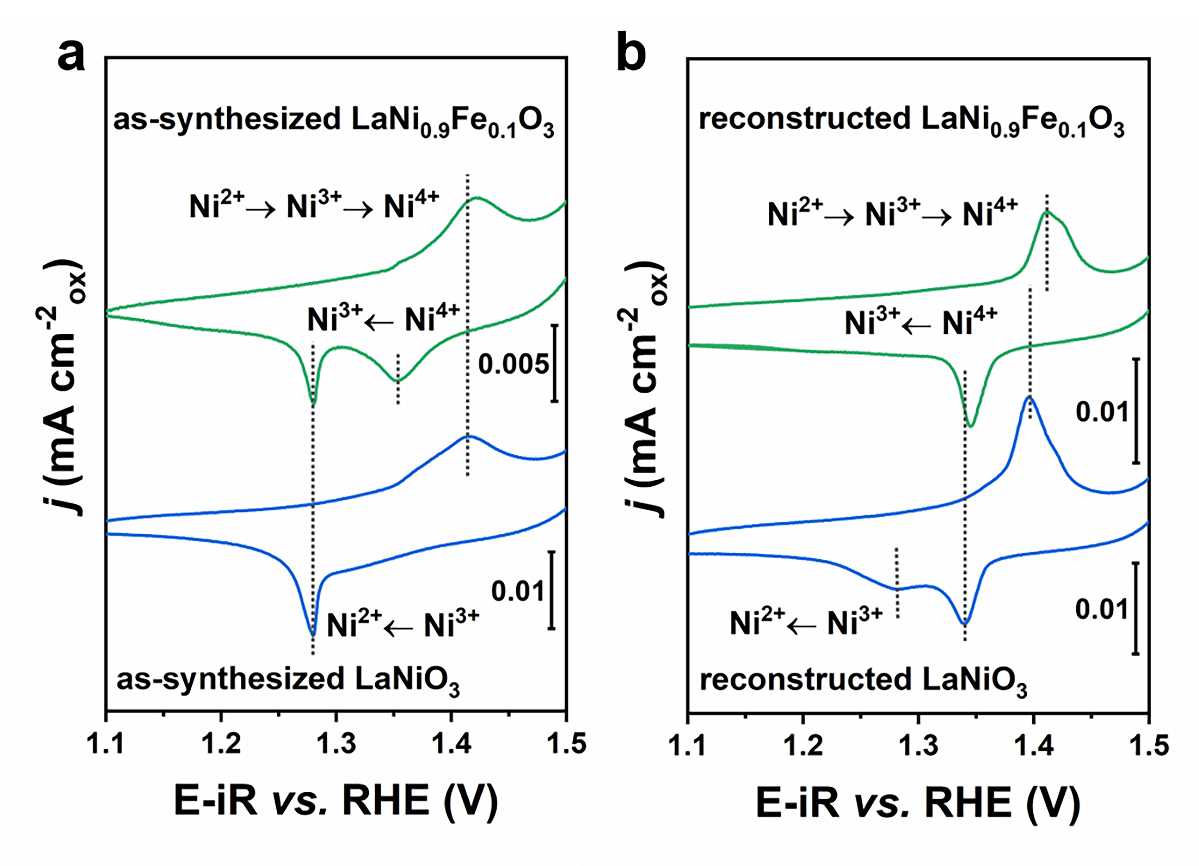
**

**Fig. S19** Magnified CV showing the pre-OER redox peaks features from 1.1 to 1.5 V (vs. RHE) for (**a**) as-synthesized LaNiO_3_ and LaNi_0.9_Fe_0.1_O_3_ and (**b**) reconstructed LaNiO_3_ and LaNi_0.9_Fe_0.1_O_3._

**
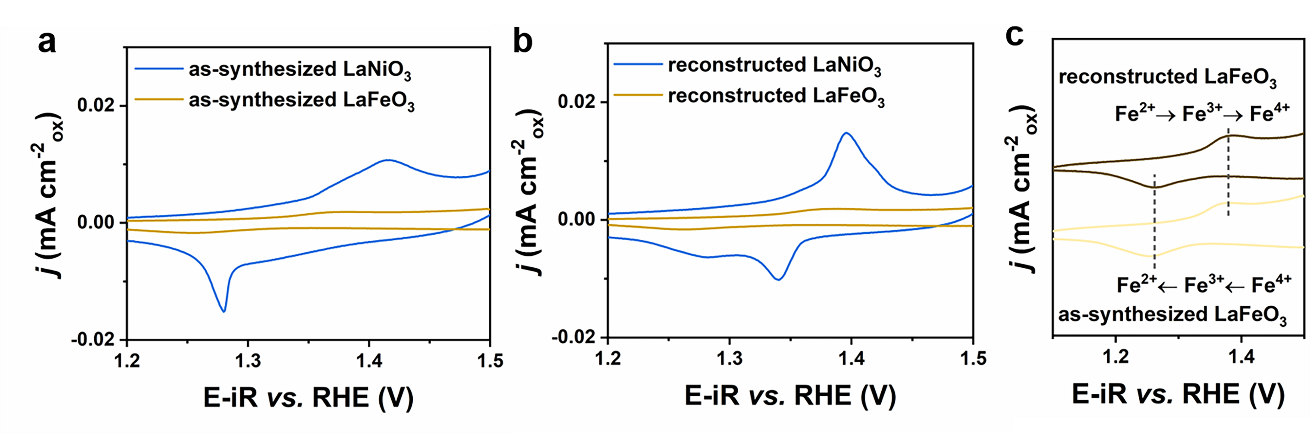
**

**Fig. S20** Magnified CV from 1.2 to 1.5 V (vs. RHE) of LaNiO_3_ and LaFeO_3_ oxides for the (**a**) as-synthesized samples and (**b**) reconstructed samples, which are overlaid for direct comparison. (**c**) Evolutive redox behavior of LaFeO_3_ in 1.0 M KOH at 10 mV s^-1^ between 1.1 and 1.5 V (vs. RHE).

**
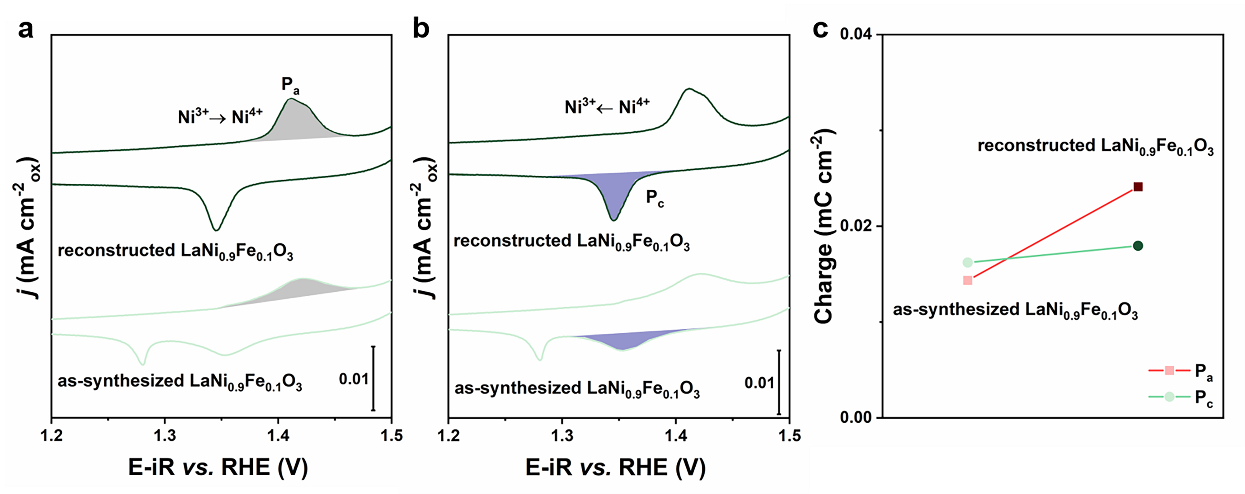
**

**Fig. S21** (**a, b**) Evolutive CV for as-synthesized LaNi_0.9_Fe_0.1_O_3_ and reconstructed LaNi_0.9_Fe_0.1_O_3_ in 1.0 M KOH at 10 mV s^-1^. The anodic and cathodic peak positions (Δ*E*_p,a_ and Δ*E*_p,c_) are labeled with gray and purple in (a) and (b) respectively. (**c**) Evolution of the charge associated with anodic and cathodic peaks in 1.0 M KOH for as-synthesized LaNi_0.9_Fe_0.1_O_3_ and reconstructed LaNi_0.9_Fe_0.1_O_3_. Owing to the low content of Ni^2+^, we analyzed charge transfer based on Ni^3+^/Ni^4+^ transitions.

**
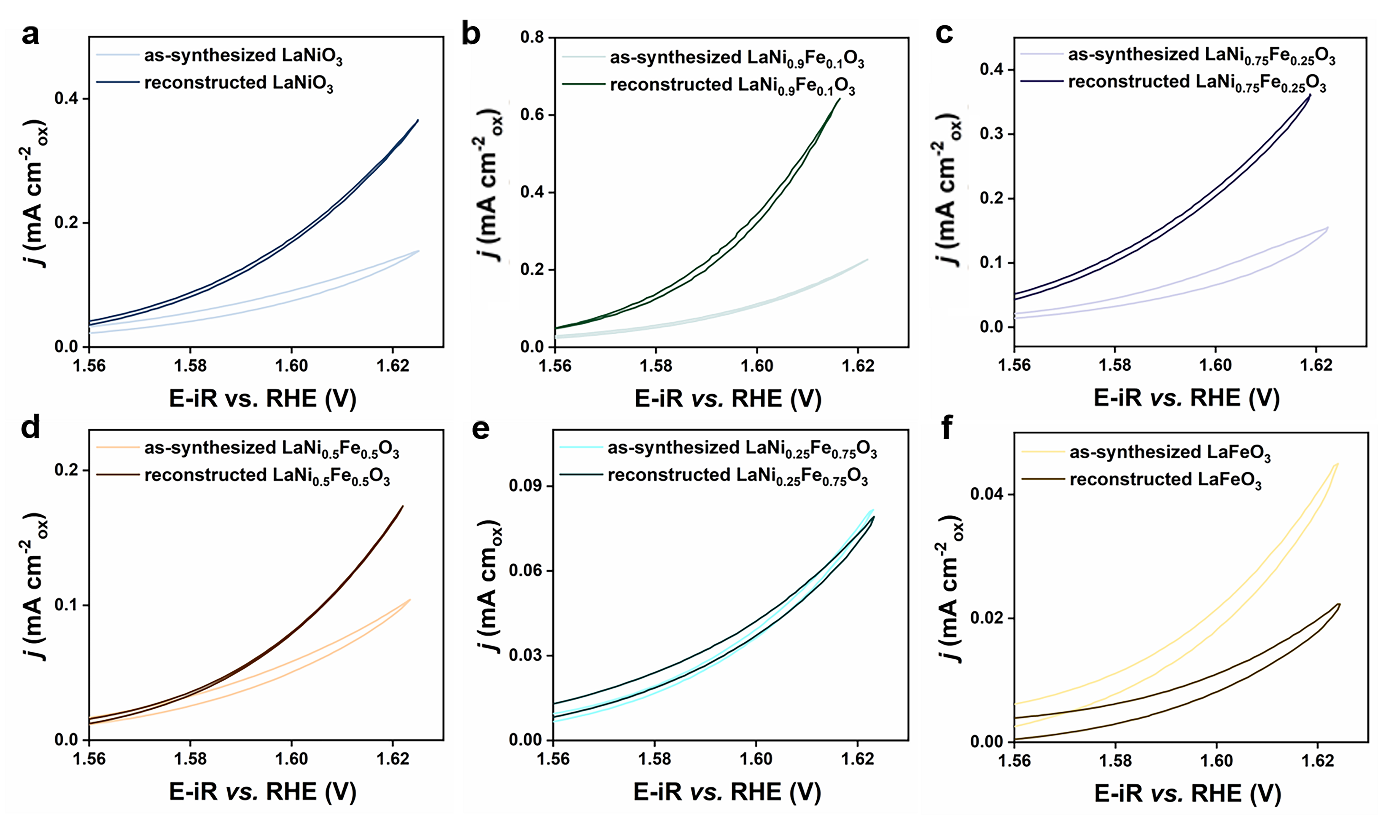
**

**Fig. S22** Evolutive CV for LaNi_1-x_Fe_x_O_3_ (x = 0.00, 0.10, 0.25, 0.50, 0.75 and 1.00) before and after reconstruction in 1.0 M KOH at 10 mV s^-1^

**
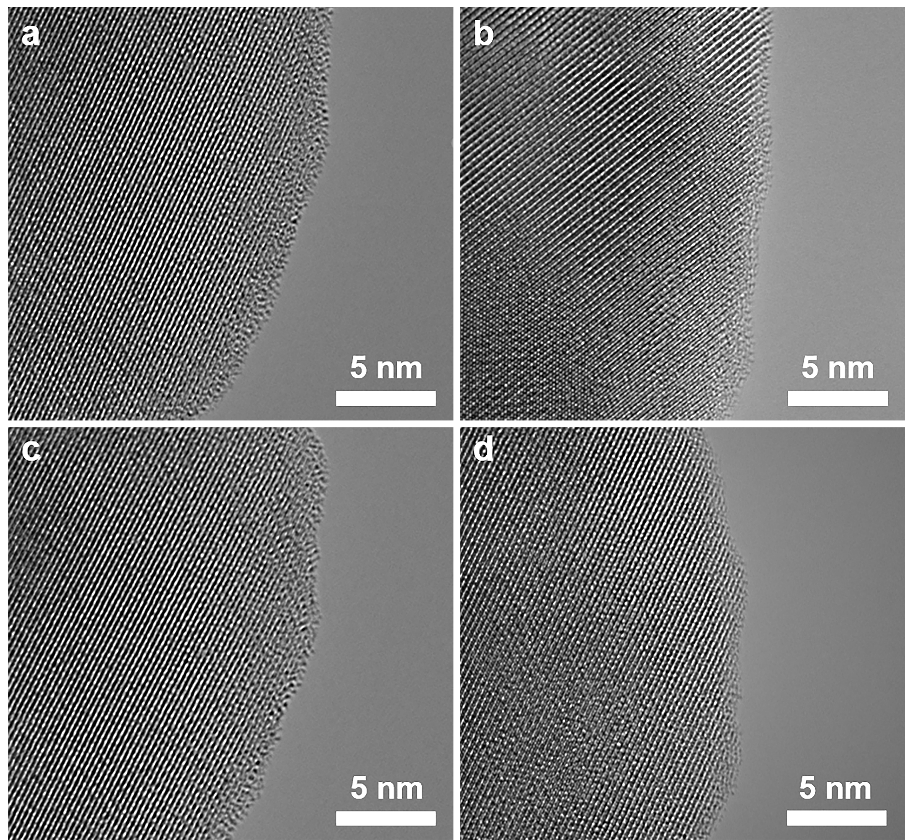
**

**Fig. S23** HRTEM images obtained from different areas, showing the surface regions of as-prepared LaNi_0.9_Fe_0.1_O_3_ oxide.


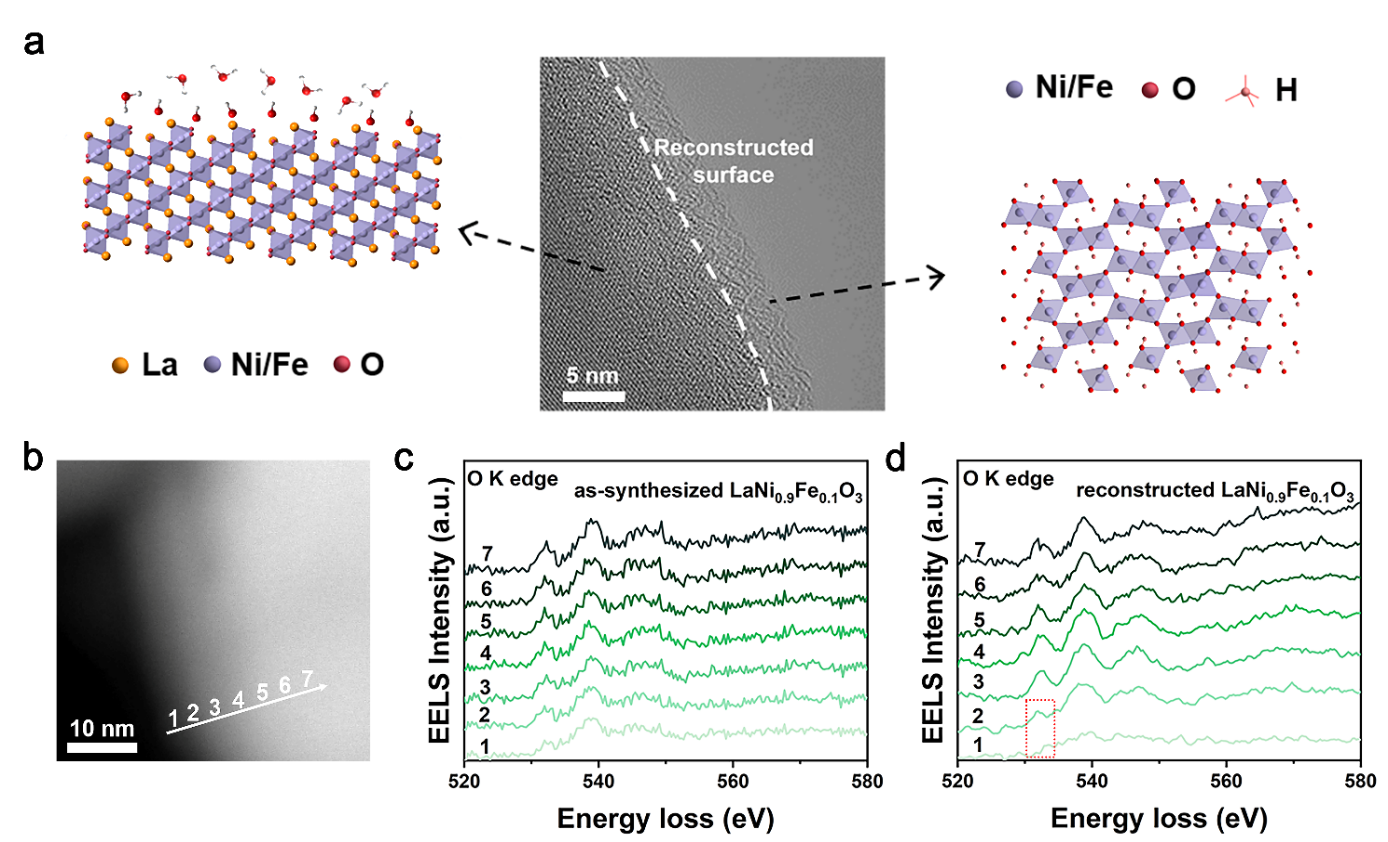


**Fig. S24** HRTEM images of reconstructed LaNi_0.9_Fe_0.1_O_3_ and the schematic diagrams of the crystal structures of the inner and outer regions.

**
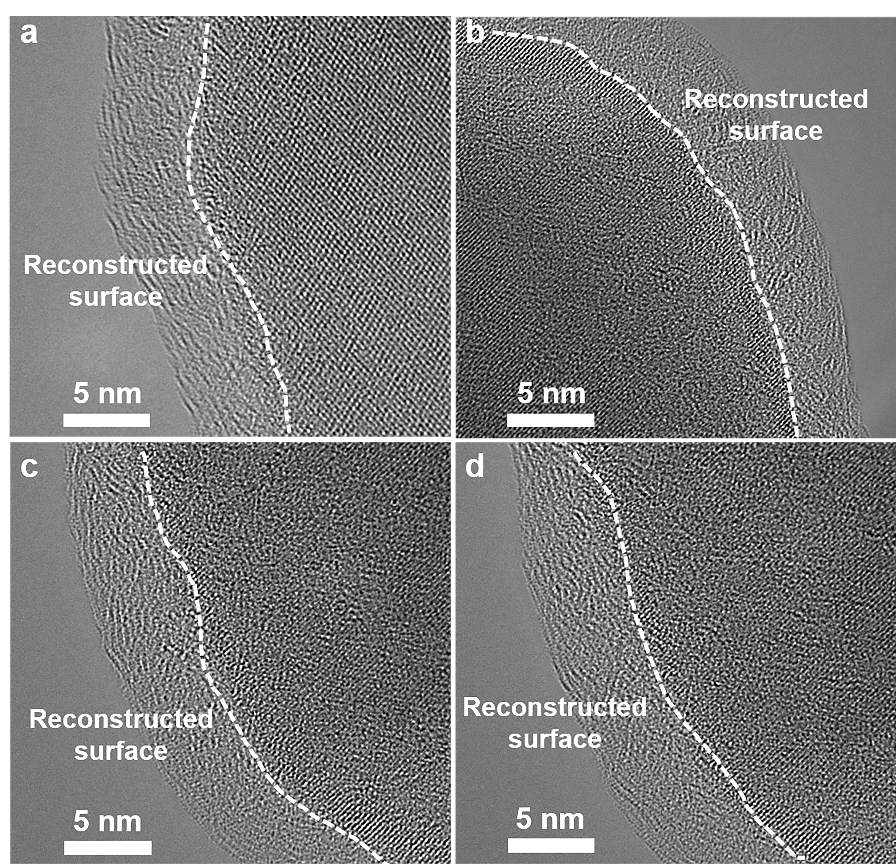
**

**Fig. S25** HRTEM images obtained from different areas, showing the surface regions of the reconstructed LaNi_0.9_Fe_0.1_O_3_ oxide.

**
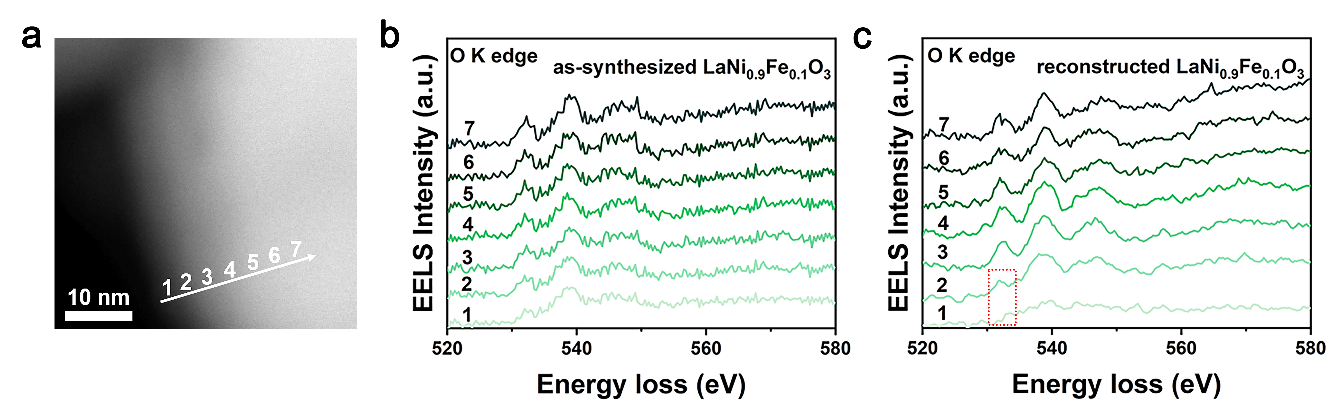
**

**Fig. S26** (**a**) STEM image of reconstructed LaNi_0.9_Fe_0.1_O_3_ oxide along the scanning pathway. (**b**) O K-edge EELS spectra along the inward scanning pathway from Point 1 to Point 7 (Fig. 13) of the as-synthesized LaNi_0.9_Fe_0.1_O_3_ oxide. (**c**) O K-edge EELS spectra of the reconstructed LaNi_0.9_Fe_0.1_O_3_ oxide along the scanning pathway from Point 1 to Point 7.

**

**

**Fig. S27** FTIR spectra of LaNi_0.9_Fe_0.1_O_3_ catalyst in the pristine state and after the activation process.

**
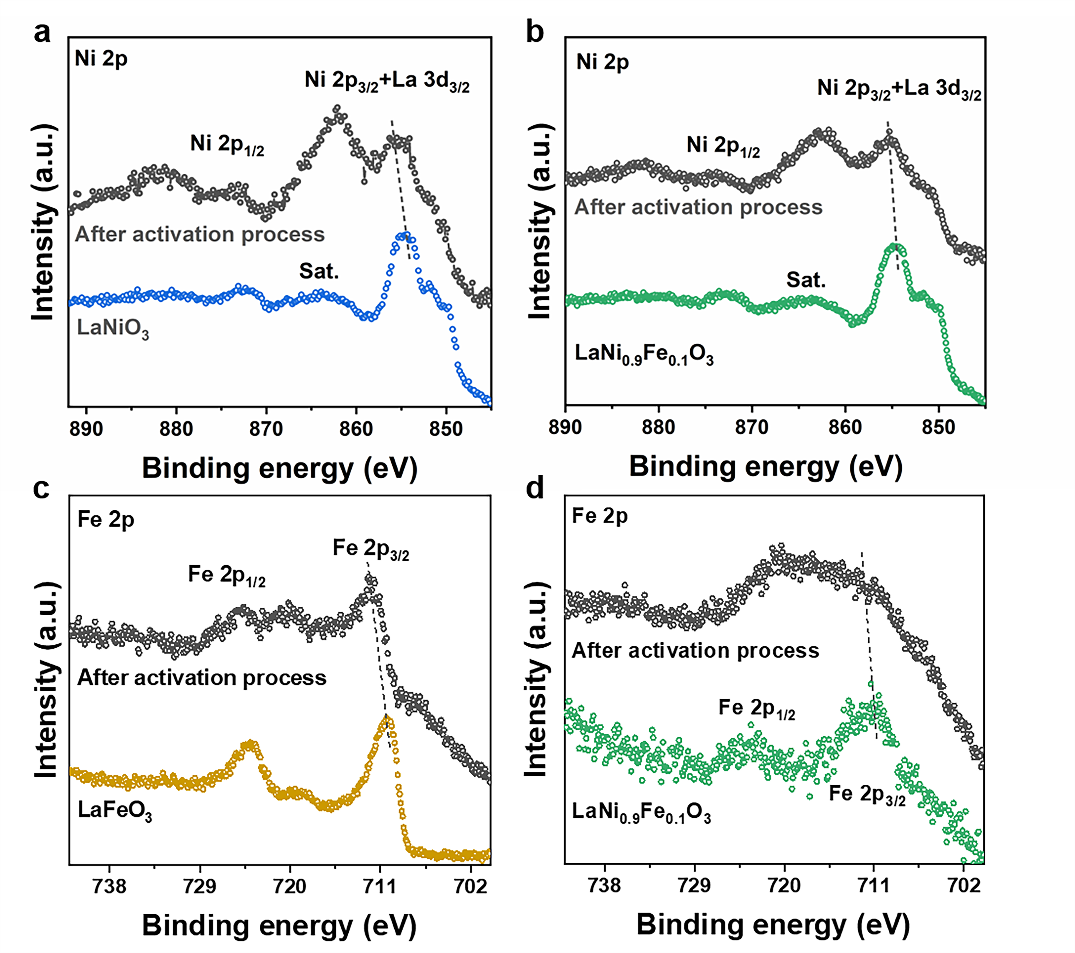
**

**Fig. S28** XPS spectra of Ni 2p for (**a**) LaNiO_3_ and (**b**) LaNi_0.9_Fe_0.1_O_3_ catalysts in the pristine state and after the activation process. XPS spectra of Fe 2p for (**c**) LaFeO_3_ and (**d**) LaNi_0.9_Fe_0.1_O_3_ catalysts in the pristine state and after the activation process.

**
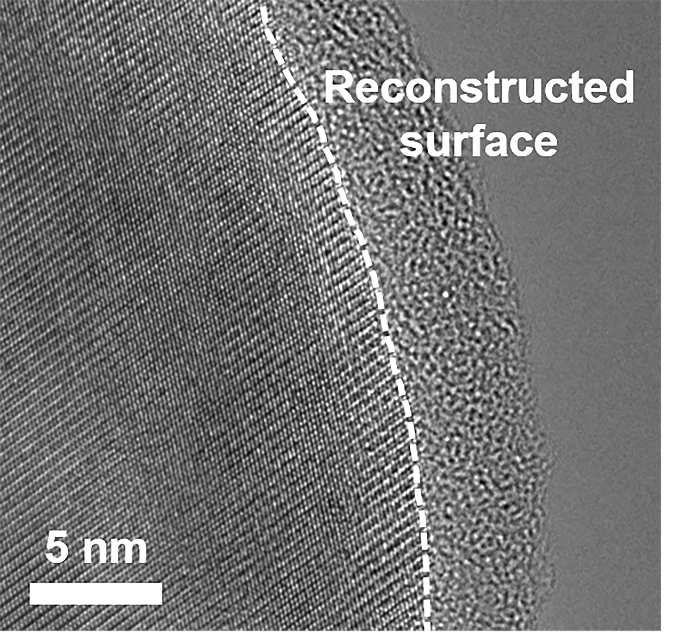
**

**Fig. S29** HRTEM images of the reconstructed LaNi_0.9_Fe_0.1_O_3_ oxide after 100 h CP measurement. The reconstruction surface of reconstructed LaNi_0.9_Fe_0.1_O_3_ oxide is found only at a limited depth (~5 nm), and the reconstructed surface is quite stable during the subsequent 100 h CP test (with a similar depth of ~5 nm), demonstrating stable surface chemistry after reconstruction.

**
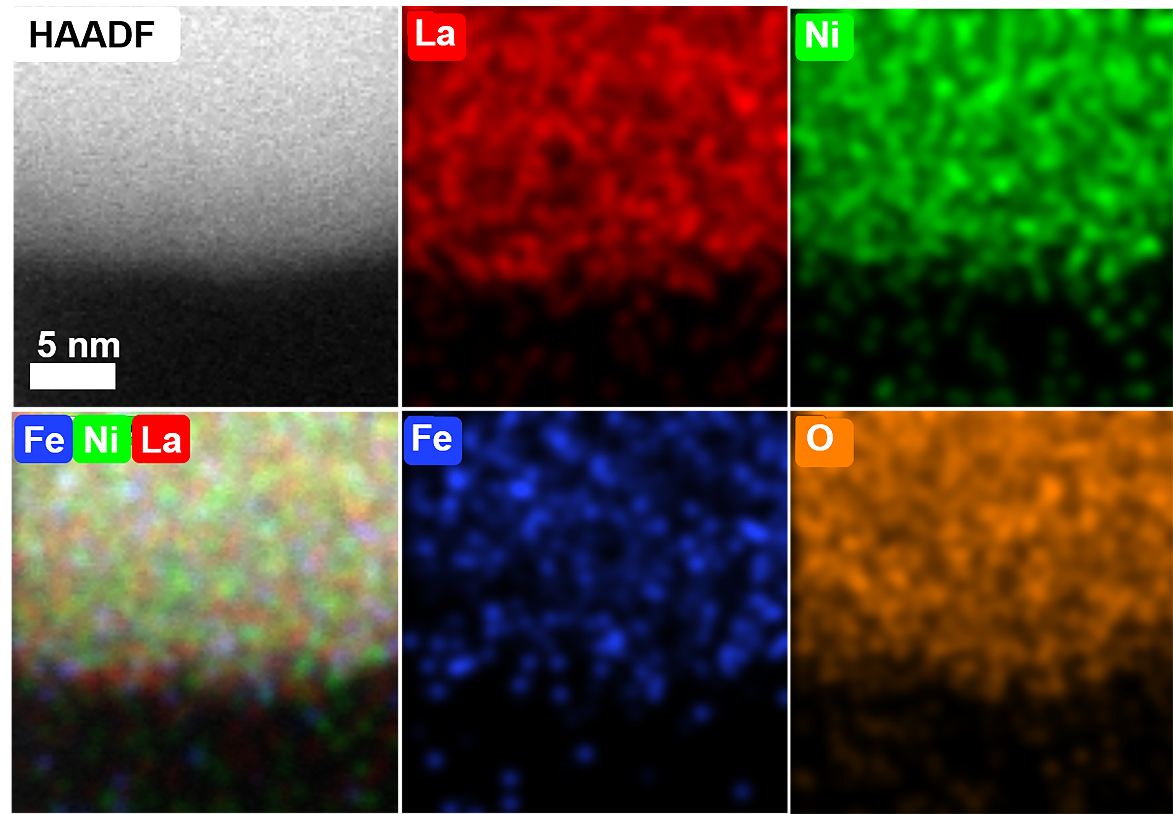
**

**Fig. S30** EDS elemental mapping of the reconstructed LaNi_0.9_Fe_0.1_O_3_ oxide after 100 h CP test.

**
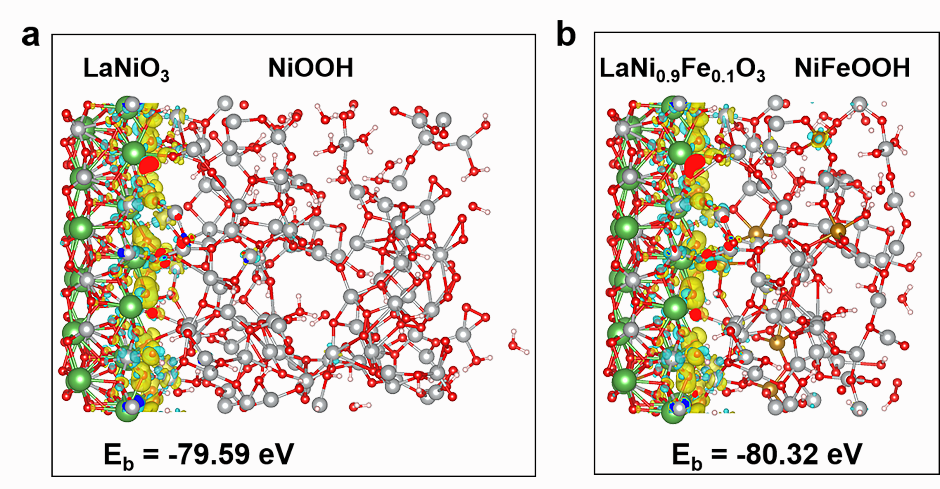
**

**Fig. S31** The deformation charge density and the corresponding E_b_ (binding energy) value of LaNiO_3_/NiOOH and LaNi_0.9_Fe_0.1_O_3_/Ni_0.9_Fe_0.1_OOH.

**
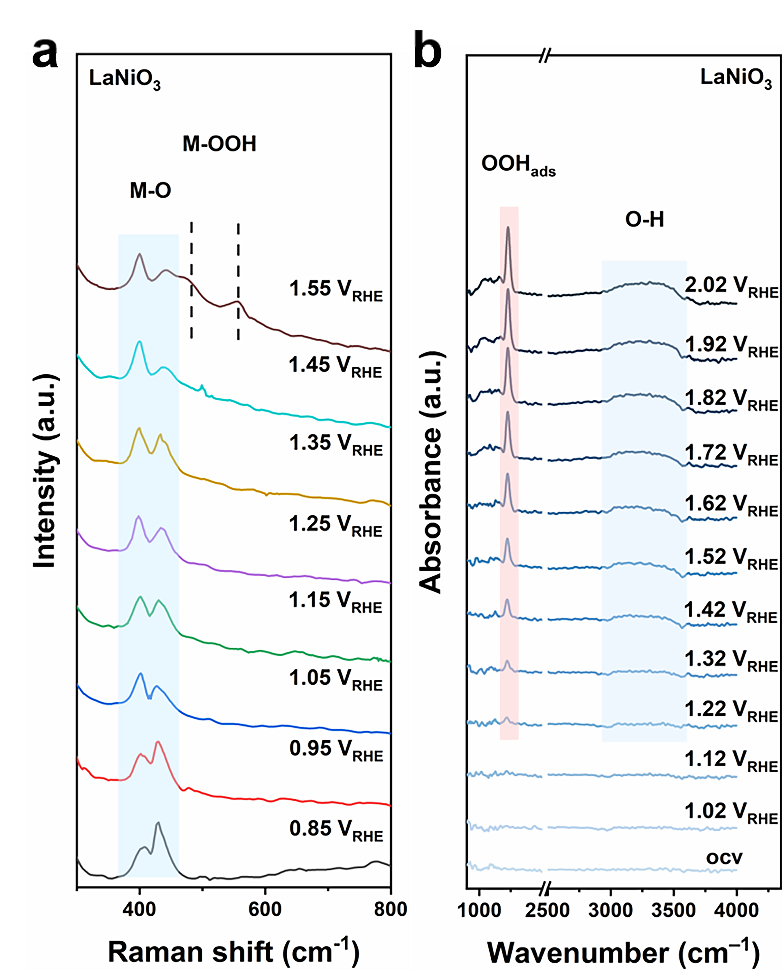
**

**Fig. S32** (**a**) Potential-dependent in-situ Raman spectra of LaNiO_3_. (**b**) In-situ ATR-IR spectra recorded during the multi-potential steps for LaNiO_3_.

**
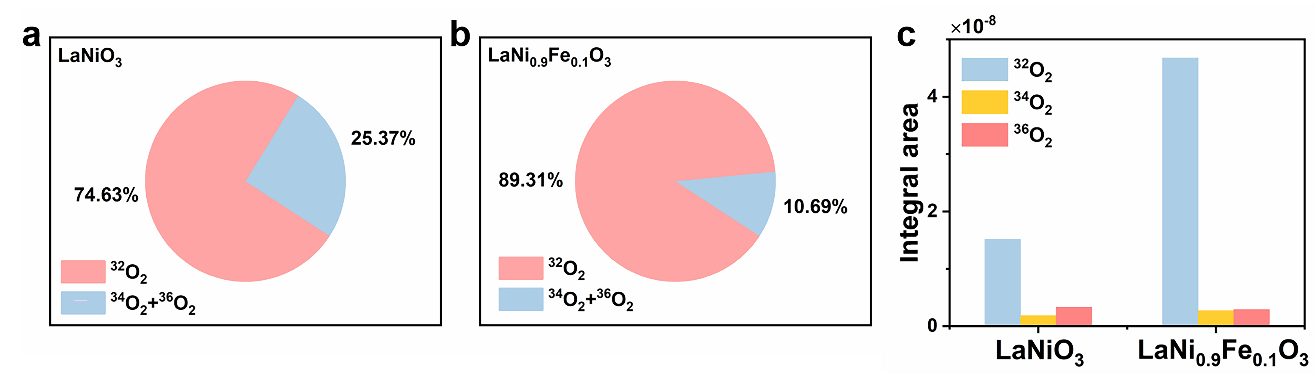
**

**Fig. S33** The percentages of ^16^O_2_ and ^18^O_2_ + ^16^O^18^O of (**a**) LaNiO_3_ and (**b**) LaNi_0.9_Fe_0.1_O_3_ oxide. (**c**) A histogram of the distribution of isotopes of O_2_ molecules obtained from integrating the area under the relevant peaks of DEMS spectra [S14].

Table S1 Elemental compositions of Ni and Fe for the as-synthesized LaNi_1-x_Fe_x_O_3_ (x = 0.00, 0.10, 0.25, 0.50, 0.75, and 1.00) oxides determined by ICP-OES

| Sample | The atomic ratio of Ni:Fe | The atomic ratio of Fe/(Ni+Fe) |
| --- | --- | --- |
| LaNiO_3_ | - | - |
| LaNi_0.9_Fe_0.1_O_3_ | 8.95:1.00 | 0.10 |
| LaNi_0.75_Fe_0.25_O_3_ | 3.14:1.00 | 0.24 |
| LaNi_0.5_Fe_0.5_O_3_ | 0.95:1.00 | 0.51 |
| LaNi_0.25_Fe_0.75_O_3_ | 0.32:1.00 | 0.76 |
| LaFeO_3_ | - | - |

Table S2 The specific surface area of the as-synthesized LaNi_1-x_Fe_x_O_3_ estimated from Brunauer-Emmett-Teller (BET) theory

| Sample | BET Area (m^2^ g^-1^) | Area (cm^2^) |
| --- | --- | --- |
| LaNiO_3_ | 3.7592 | 1.8796 |
| LaNi_0.9_Fe_0.1_O_3_ | 4.2752 | 2.1376 |
| LaNi_0.75_Fe_0.25_O_3_ | 3.4589 | 1.72945 |
| LaNi_0.5_Fe_0.5_O_3_ | 5.912 | 2.956 |
| LaNi_0.25_Fe_0.75_O_3_ | 5.7688 | 2.8844 |
| LaFeO_3_ | 7.809 | 3.9045 |

**Table S3** The redox charge of LaNi_0.9_Fe_0.1_O_3_ perovskite oxide calculating before and after the reconstruction process

|  | q_a_ (mC cm^-2^) | q_c_ (mC cm^-2^) |
| --- | --- | --- |
| Before reconstruction | 0.014 | 0.016 |
| After reconstrution | 0.024 | 0.018 |

**Table S4** Comparison of Ni and Fe ratios in as-synthesized and reconstructed LaNi_0.9_Fe_0.1_O_3_ oxides

| Sample | The atomic ratio of Ni:Fe | The atomic ratio of Fe/(Ni+Fe) |
| --- | --- | --- |
| s-synthesized LaNi_0.9_Fe_0.1_O_3_ | 8.95:1.00 | 0.10 |
| reconstructed LaNi_0.9_Fe_0.1_O_3_ | 8.75:1.00 | 0.10 |

**Supplementary References**

1. P. Chakthranont, J. Kibsgaard, A. Gallo, J. Park, M. Mitani, et al., Effects of gold substrates on the intrinsic and extrinsic activity of high-Loading nickel-based oxyhydroxide oxygen evolution catalysts. ACS Catal. **7**, 5399 (2017). <https://doi.org/10.1021/acscatal.7b01070>
2. L. Trotochaud, S. L Young, J. K. Ranney, S. W. Boettcher, Nickel–iron oxyhydroxide oxygen-evolution electrocatalysts: The role of intentional and incidental iron incorporation. J. Am. Chem. Soc. **136**, 6744 (2014). <https://doi.org/10.1021/ja502379c>
3. F. Song, X. Hu, Exfoliation of layered double hydroxides for enhanced oxygen evolution catalysis. Nat. Commun*.* **5**, 4477 (2014). <https://doi.org/10.1038/ncomms5477>
4. A. S. Kelsey, R. Marcel, H. B. Hong, S.-H. Yang, Orientation-dependent oxygen evolution activities of rutile IrO_2_ and RuO_2._ J. Phys. Chem. Lett. **5**, 1636 (2014). <https://doi.org/10.1021/jz500610u>
5. L. D. Burke, O. J. Murphy, Cyclic voltammetry as a technique for determining the surface area of RuO_2_ electrodes. J. Electroanal. Chem. **96**, 19 (1979). <https://doi.org/10.1016/S0022-0728(79)80299-5>
6. Y. Sun, R. Li, X. Chen, J. Wu, Y. Xie, et al., A-Site management prompts the dynamic reconstructed active phase of perovskite oxide OER catalysts. Adv. Energy Mater. **11**, 2003755 (2021). <https://doi.org/10.1002/aenm.202003755>
7. H. Wang, J. Wang, Y. Pi, Q. Shao, Y. Tan, et al. Double perovskite LaFe_x_Ni_1−x_O_3_ nanorods enable efficient oxygen evolution electrocatalysis. Angew. Chem. Int. Ed. **58**, 2316 (2019). <https://doi.org/10.1002/anie.201812545>
8. J. Wang, S.-J. Kim, J. Liu, Y. Gao, S. Choi, J. Han, et al. Redirecting dynamic surface restructuring of a layered transition metal oxide catalyst for superior water oxidation. Nat. Catal. **4**, 212 (2014). <https://doi.org/10.1038/s41929-021-00578-1>
9. X. Wang, K. Huang, L. Yuan, S. Li, W. Ma, et al., Molten salt flux synthesis, crystal facet design, characterization, electronic structure, and catalytic properties of perovskite cobaltite. ACS Appl. Mater. Interfaces **10**, 28219 (2018). <https://doi.org/10.1021/acsami.8b08621>
10. C. Kuai, Z. Xu, C. Xi, A. Hu, Z. Yang, et al. Phase segregation reversibility in mixed-metal hydroxide water oxidation catalysts. Nat. Catal*.* **3**, 743 (2020). <https://doi.org/10.1038/s41929-020-0496-z>
11. C. Kuai, C. Xi, A. Hu, Y. Zhang, Z. Xu, et al. Revealing the dynamics and roles of iron incorporation in nickel hydroxide water oxidation catalysts J. Am. Chem. Soc*.* **143**, 18519 (2021). <https://doi.org/10.1021/jacs.1c07975>
12. H. Wang, J. Qi, N. Yang, W. Cui, J. Wang, et al. Dual-defects adjusted crystal-field splitting of LaCo_1−x_Ni_x_O_3−δ_ hollow multishelled structures for efficient oxygen evolution. Angew. Chem. Int. Ed. **59**, 1969 (2020). <https://doi.org/10.1002/anie.202007077>
13. Y. Duan, S. Sun, Y. Sun, S. Bo, X. Chi, et al. Mastering surface reconstruction of metastable spinel oxides for better water oxidation. Adv. Mater. **31**, 1807898 (2019). <https://doi.org/10.1002/adma.201807898>
14. B. Wu, S. Gong, Y. Lin, T. Li, A. Chen, et al. A Unique NiOOH@FeOOH heteroarchitecture for enhanced oxygen revolution in saline water. Chen. Adv. Mater. **34**, 2108619 (2022). <https://doi.org/10.1002/adma.202108619>
